# Exploring the Mutated Kinases for Chemoenzymatic Synthesis of *N*^4^-Modified Cytidine Monophosphates

**DOI:** 10.3390/molecules29163767

**Published:** 2024-08-09

**Authors:** Martyna Koplūnaitė, Kamilė Butkutė, Jonita Stankevičiūtė, Rolandas Meškys

**Affiliations:** Department of Molecular Microbiology and Biotechnology, Institute of Biochemistry, Life Sciences Center, Vilnius University, Sauletekio Av. 7, LT-10257 Vilnius, Lithuania; kamile.butkute@bchi.stud.vu.lt (K.B.); jonita.stankeviciute@bchi.vu.lt (J.S.)

**Keywords:** nucleoside 5′-monophosphates, nucleotides, nucleoside analogues, deoxynucleoside kinase, deoxycytidine kinase, mutagenesis, chemical synthesis, biocatalysis

## Abstract

Nucleosides, nucleotides, and their analogues are an important class of molecules that are used as substrates in research of enzymes and nucleic acid, or as antiviral and antineoplastic agents. Nucleoside phosphorylation is usually achieved with chemical methods; however, enzymatic phosphorylation is a viable alternative. Here, we present a chemoenzymatic synthesis of modified cytidine monophosphates, where a chemical synthesis of novel *N*^4^-modified cytidines is followed by an enzymatic phosphorylation of the nucleosides by nucleoside kinases. To enlarge the substrate scope, multiple mutant variants of *Drosophila melanogaster* deoxynucleoside kinase (*Dm*dNK) (EC:2.7.1.145) and *Bacillus subtilis* deoxycytidine kinase (*Bs*dCK) (EC:2.7.1.74) have been created and tested. It has been determined that certain point mutations in the active sites of the kinases alter their substrate specificities noticeably and allow phosphorylation of compounds that had been otherwise not phosphorylated by the wild-type *Dm*dNK or *Bs*dCK.

## 1. Introduction

Nucleoside and nucleotide analogues are an invaluable asset in the fields of molecular biology, biotechnology, and pharmacology. Modified nucleosides and their phosphates are employed in enzyme or nucleic acid research, and some are key building blocks of modern RNA vaccines [1,2,3,4,5]. In addition, certain nucleoside analogues are known to possess antiviral or anticancer properties [6,7]. Due to the immense interest in such compounds, the need for efficient nucleotide preparation is ever-growing. Generally, nucleoside phosphorylation is achieved utilizing chemical methods; however, they are not without drawbacks and must be chosen with caution depending on the target nucleoside. Synthetic approaches usually rely on extremely reactive phosphochloride compounds [8]. Such reactions are infamous for their harsh conditions, the usage of harmful reagents, and the need for protective group chemistry. A welcome alternative to chemical phosphorylation is the use of phosphorylating enzymes. Nucleoside phosphorylation can be achieved by the help of nucleoside kinases [9,10,11,12,13]. Some examples are nucleoside kinases such as *Homo sapiens* uridine–cytidine kinase (*Hs*UCK), *Homo sapiens* adenosine kinase (*Hs*AK), *Escherichia coli* guanosine–inosine kinase, and deoxynucleoside kinases such as *Homo sapiens* deoxycytidine kinase (*Hs*dCK), herpes simplex virus 1 thymidine kinase (HSV-1 TK), *Drosophila melanogaster* deoxynucleoside kinase (*Dm*dNK), and *Bacillus subtilis* deoxycytidine kinase (*Bs*dCK) [14,15,16,17,18,19,20].

**Figure 1:**
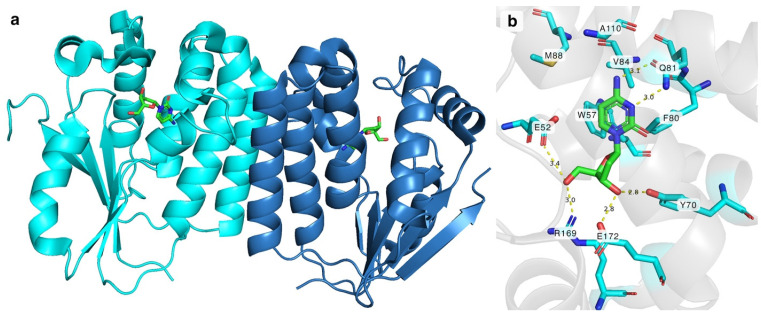
(**a**) A dimer of *Dm*dNK with 2′-deoxycytidine molecules (green) bound in the active centres. Each monomer is coloured in different shades of blue. (**b**) Interactions between 2′-deoxycytidine (green) and amino acid residues (cyan) in the active site of deoxynucleoside kinase. F114 is omitted for a clearer view. Yellow dashed lines represent hydrogen bonds between the nucleoside and the residues. PDB ID: 1j90.

*Dm*dNK (EC:2.7.1.145) is a multisubstrate kinase that phosphorylates all canonical deoxy- and ribonucleosides with a biased affinity towards pyrimidine nucleobase-bearing deoxyribonucleosides [21]. In addition to canonical nucleosides, nucleoside analogues with various modifications at nucleobase or sugar are also acceptable substrates to the kinase [22,23,24]. Crystallographic structures have been solved with different substrates, including but not limited to 2′-deoxycytidine, 2′-deoxythymidine, 5-fluoro-2′-deoxycytidine, (*E*)-5-(2-bromovinyl)-2′-deoxyuridine, and 2′-deoxythymidine 5′-triphosphate [25,26,27]. The active form of the enzyme is a homodimer (Figure 1a). The monomer of *Dm*dNK (29 kDa) consists of eight α-helices and a central five-strand parallel β-sheet. Crystallographic data of *Dm*dNK with a bound 2′-deoxycytidine has revealed that the active site is lined with hydrophobic residues (Figure 1b). When a substrate is bound, the nucleobase is stabilized by aromatic residues W57, F80, and F114. In addition, Q81 interacts with *N*^3^ and *N*^4^ atoms of the nucleobase. Polar residues Y70 and E172 form hydrogen bonds with the 3′-hydroxy group of the ribose moiety, and E52, R169, and a water molecule anchors the 5′-OH in place. The E52 residue together with R105 acts as a reaction initiator by deprotonating the 5′-hydroxygroup of the ribose. The deep cavity, lined with hydrophobic residues V84, M88, and A110, and located near the C5 position of cytosine moiety, might offer an explanation as to why the kinase accepts such a variety of substrates with modifications at positions *N*^4^ or C5 [25].

**Figure 2:**
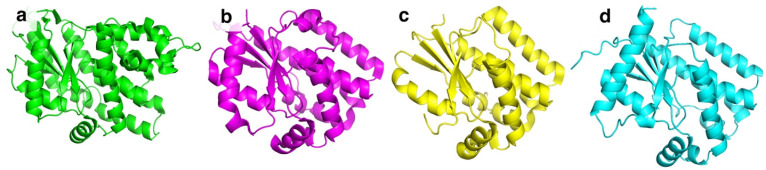
Tertiary structures of non-TK1-like family nucleoside kinases. (**a**) HSV-1 TK monomer. PDB ID: 4IVP. (**b**) *Hs*dCK monomer. PDB ID: 1p61. (**c**) *Dm*dNK monomer. PDB ID: 1j90. (**d**) *Bs*dCK monomer. Model generated by ColabFold v1.5.5.

BsdCK (EC:2.7.1.74) is a nucleoside kinase that belongs to the non-TK1-like kinase family together with HSV-1 TK, *Hs*dCK, and *Dm*dNK [10]. All kinases belonging to this family share a similar fold (Figure 2a–d) and are found as homodimers. In contrast to the aforementioned kinases, *Bs*dCK has not been studied thoroughly and its crystal structure has yet to be determined. The mass of its monomer is 25 kDa. Sequence analysis has revealed that it has a 25.6% identity to *Dm*dNK and a 25.3% identity to *Hs*dCK. The main substrates of *Bs*dCK are nucleosides of cytosine and adenine [28]. The specificity toward sugar moiety is quite lenient. Substrates bearing ribose, 2′-deoxyribose, and arabinose moieties are accepted, although deoxyribonucleosides are favoured. As discovered in our previous study, the substrate specificity of *Bs*dCK is not as limited as initially believed [23]. Furthermore, *Bs*dCK phosphorylates all canonical ribo- and 2′-deoxyribonucleosides; however, phosphorylation of thymidine is poor. The kinase also accepts a variety of pyrimidine nucleoside analogues bearing modifications at the nucleobase such as *N*^4^-acetyl-2′-deoxycytidine, 5-fluorocytidine, *N*^1^-methylpseudouridine, or 2-thiouridine. Moreover, modifications of the sugar moiety are also tolerated. Some examples include 2′-*O*-methyluridine, 2′,3′-*O*-isopropylidineuridine, 2′-*N*-acetyl-2′-amino-2′-deoxyuridine, and 2′,3′-dideoxyuridine [23].

Even though the mild reaction conditions and high regioselectivity make enzymatic nucleoside phosphorylation a compelling alternative to chemical nucleotide synthesis, it is far from perfect. Enzymatic phosphorylation is limited by the substrate specificity of the utilized enzymes and is often plagued with low to moderate product yields.

This work is a continuation of our previous study where we applied *Dm*dNK and *Bs*dCK for the phosphorylation of base- and sugar-modified nucleosides and discovered that even though the kinases possess a broad substrate specificity, a number of modified nucleosides remained non-phosphorylated [23]. To expand the substrate scope, we have created 16 *Dm*dNK and 3 *Bs*dCK mutant variants and tested them towards various *N*^4^-modified cytidine nucleosides.

## 2. Results and Discussion

The aim of our study was to increase the activities of *Dm*dNK and *Bs*dCK towards nucleobase-modified cytidine nucleosides. As we have determined in our previous investigation of wild-type *Dm*dNK and *Bs*dCK, the two kinases can phosphorylate a variety of pyrimidine analogues with small to medium-sized nucleobase or sugar modifications [23]. In the current study, we have focused on cytidine and 2′-deoxycytidine nucleosides bearing modifications at the *N*^4^-position.

### 2.1. Substrates Exploited in the Study

**Figure 3:**
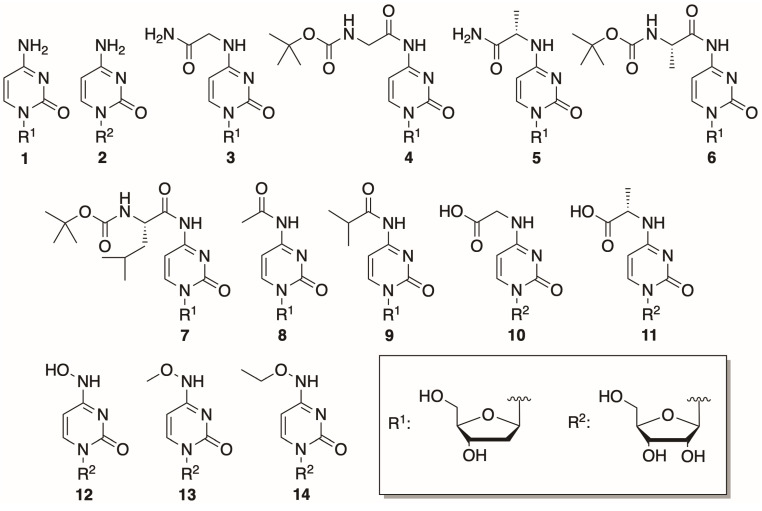
Structures of the canonical and *N*^4^-modified nucleosides used in the study.

In this study, 14 cytosine nucleosides were chosen to be tested as phosphorylation substrates. The structures of the nucleosides are provided in Figure 3. Two canonical nucleosides, 2′-deoxycytidine and cytidine, were picked to assess the activity of the mutant variants since they are known to be efficiently phosphorylated by both *Dm*dNK-WT and *Bs*dCK-WT [20,21]. The remaining 12 nucleosides were chosen to be as diverse as possible and had modifications at the *N*^4^-position of the cytosine nucleobase, that ranged from small to bulky, from polar to hydrophobic. Nucleosides **1**, **2**, **8**, and **9** were obtained commercially, while the rest were synthesized in our laboratory. Preparation of the *N*^4^-amino acid-modified nucleosides (**3**–**7**) was described previously [29]. Syntheses of the acidic nucleosides (**10** and **11**), and *N*^4^-hydroxy- (**12**) and *N*^4^-alkoxy-modified cytidines (**13** and **14**) are provided below.

#### 2.1.1. Synthesis of *N*-[1-(β-D-Ribofuranosyl)-2-oxo-4-pyrimidinyl]-amino Acids

**Scheme 1:**
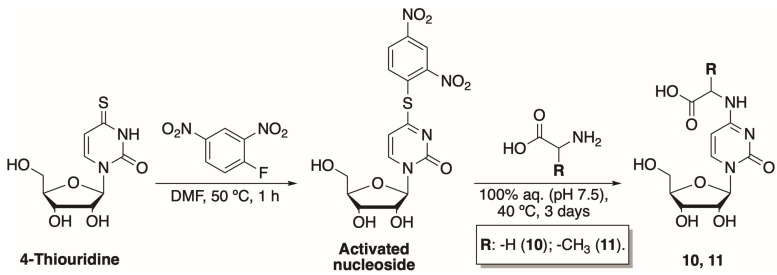
Preparation of *N*-[1-(β-D-ribofuranosyl)-2-oxo-4-pyrimidinyl]-amino acids.

To further investigate the influence of the modifications at *N*^4^-position on nucleoside phosphorylation, we have synthesized two *N*-[1-(β-D-ribofuranosyl)-2-oxo-4-pyrimidinyl]-amino acids. These compounds are analogues to our previously synthesized *N*^4^-amino acid-modified 2′-deoxycytidines [29]. The newly synthesized compounds had exposed carboxyl instead of amide functional groups. The two-step synthesis of *N*-[1-(β-D-ribofuranosyl)-2-oxo-4-pyrimidinyl]-amino acids has been adapted from Ohashi et al. (Scheme 1) [30]. Treatment of commercially available 4-thiouridine with 2,4-dinitrofluorbenzene in *N*,*N*-dimethylformamide (DMF) for 1 h at 50 °C resulted in an activated nucleoside. The compound was purified using normal phase column chromatography and obtained in an overall yield of 24%.

The activated nucleoside was then treated with the corresponding amino acid (glycine or alanine) at 40 °C using 50 mM potassium phosphate buffer (pH 7.5) as a solvent. Products **10** and **11** were purified using two different chromatographic techniques. Hence, *N*-[1-(β-D-ribofuranosyl)-2-oxo-4-pyrimidinyl]-glycine (**10**) was purified by ion-exchange chromatography, while *N*-[1-(β-D-ribofuranosyl)-2-oxo-4-pyrimidinyl]-L-alanine (**11**) was separated using reversed-phase and hydrophilic interaction liquid chromatography methods. Both products were obtained in 13% yields.

#### 2.1.2. Synthesis of *N*^4^-Hydroxy- and *N*^4^-Alkoxycytidines

**Scheme 2:**
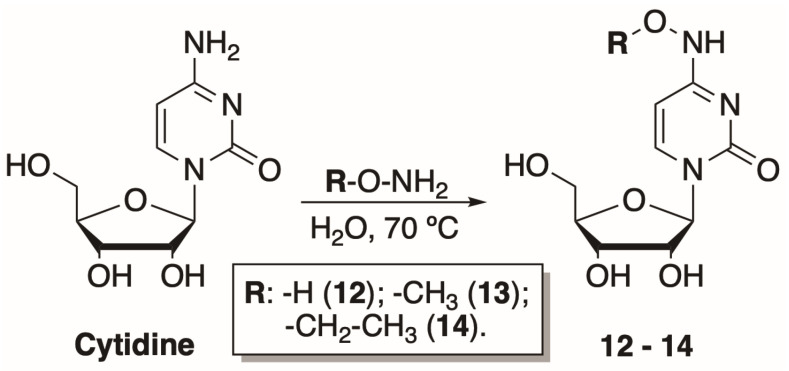
Preparation of *N*^4^-hydroxy- and *N*^4^-alkoxycytidines.

To explore how the bulkiness of the *N*^4^ modifications of the cytosine nucleobase affects the phosphorylation efficiency, three additional cytidine nucleoside analogues were synthesized. The preparation of *N*^4^-alkoxycytidines was based on *N*^4^-hydroxycytidine synthesis described by Paymode et al. [31]. The reaction between cytidine and amine hydrochloride salt in water at 70 °C resulted in corresponding hydroxy-, methoxy-, and ethoxy- modifications at the *N*^4^-position of cytidine (**12**–**14**) (Scheme 2). The products were purified by normal phase chromatography and obtained in a moderate 56–76% yield.

### 2.2. Synthesis and Application of the Mutant Kinases for the Phosphorylation of Canonical and N^4^-Modified Cytidine Nucleosides

Site-directed mutagenesis was carried out for the creation of mutant *Dm*dNK and *Bs*dCK variants, which were generated with histidine tags at the *N*-termini and applied for phosphorylation of nucleosides without further removal of the tags after purification. The preparation of the recombinant kinases is described in Materials and Methods. Sixteen mutant *Dm*dNK variants, carrying a single or a double mutation, were generated: *Dm*dNK-W57F, *Dm*dNK-W57V, *Dm*dNK-Q81A, *Dm*dNK-Q81A+V84G, *Dm*dNK-Q81A+M88G, *Dm*dNK-A110G, *Dm*dNK-V84A, *Dm*dNK-V84A+M88A, *Dm*dNK-V84A+A110D, *Dm*dNK-V84G, *Dm*dNK-M88A, *Dm*dNK-M88G, *Dm*dNK-M88R, *Dm*dNK-M88R+A110G, *Dm*dNK-A110D, and *Dm*dNK-A110G. Three *Bs*dCK mutants, carrying a single or a double mutation, were created: *Bs*dCK-R70M, *Bs*dCK-R70M+D93A, and *Bs*dCK-D93A. Mutant variants *Dm*dNK-Q81A+V84G, *Dm*dNK-Q81A+M88G, *Dm*dNK-Q81A+A110G, *Dm*dNK-V84G, *Dm*dNK-M88G, and *Dm*dNK-A110G were found to be unstable and precipitated after an overnight dialysis. The solubility of those proteins was partially overcome by replacing *E. coli* BL-21(DE3) with *E. coli* KRX strain for gene expression, and cultivation of the transformants at 20 °C instead of 37 °C for 20 h.

The mutants were employed for the phosphorylation of cytidine nucleosides, whose structures are provided in Figure 3 above. The conditions for the phosphorylation reactions are given in Section 3. The results are provided in Table 1 and Table 2.

### 2.3. Mutations in the Hydrophobic Cavity of DmdNK Active Site

As has been previously mentioned, in addition to canonical nucleosides, wild-type *Dm*dNK (*Dm*dNK-WT) is efficient at phosphorylating a broad range of modified pyrimidine nucleosides [21,23,32]. One of the reasons why such a wide variety of nucleoside analogues is accepted by the kinase is the voluminous cavity found in the active site of the enzyme, that allows for accommodation of cytosine nucleosides bearing modifications at C5 or *N*^4^ positions [27]. The residues that line the cavity are V84, M88, and A110. Knecht and colleagues have examined the significance of the residues by creating point mutations at the aforementioned positions [33]. It has been discovered that the replacement of V84 with alanine decreases the affinity towards deoxythymidine and makes deoxycytidine the preferred substrate of *Dm*dNK mutant variant V84A. The shift in affinity occurs due to the loss of favourable interactions between valine and the methyl group of deoxythymidine. In addition, a triple mutant *Dm*dNK-V84S+M88R+A110D, which mimics active sites of deoxyguanosine kinases (dGK), has displayed substrate preferences similar to those of dGK, and favoured deoxyguanosine over deoxycytidine as a substrate [25]. In another study, a triple mutant *Dm*dNK-V84A+M88R+A110D enabled phosphorylation of purine nucleoside analogues, 9-β-D-arabinofuranosyladenine, 9-β-D-arabinofuranosylguanine, and 2′,2′-difluorodeoxyguanosine, which were not accepted as substrates by the *Dm*dNK-WT [34].

To investigate whether modifications of the hydrophobic cavity can lead to an increased activity towards *N*^4^-modified cytosine nucleosides, we have created point mutations at the positions V84, M88, and A110, respectively. Our hypothesis was that the extra space gained by replacing these residues with smaller alanine or glycine residues would allow the entering of substrates with bulkier modifications. In total, seven single mutant variants with modifications at the hydrophobic cavity were generated. V84 was replaced with alanine (*Dm*dNK-V84A) and glycine (*Dm*dNK-V84G); M88 was replaced with alanine (*Dm*dNK-M88A), glycine (*Dm*dNK-M88G), and arginine (*Dm*dNK-M88R); A110 was replaced with aspartate (*Dm*dNK-A110D) and glycine (*Dm*dNK-A110G). In addition, three double mutants were created (*Dm*dNK-V84A+M88A, *Dm*dNK-V84A+A110D, and *Dm*dNK-M88R+A110G) to investigate whether any synergistic effects were present. The data on the activity of the developed mutants is presented in Table 1.

When tested with canonical deoxycytidine (**1**), all variants displayed similar performance to *Dm*dNK-WT and converted the compound to its monophosphate with 79–98% efficiency, with only a couple of poor performers (*Dm*dNK-V84A+A110D and *Dm*dNK-M88R-A110G), whose efficiencies reached 22–34%. Mutants *Dm*dNK-V84A, *Dm*dNK-V84A+M88A, and *Dm*dNK-A110G retained the same activities towards cytidine (**2**) as *Dm*dNK-WT and phosphorylated the compound with 65–75% efficiencies, while the activities of the remaining mutants decreased to 1–41%.

As evident from our results, replacing V84 with either alanine (*Dm*dNK-V84A) or glycine (*Dm*dNK-V84G) more than triples the activity of the kinase towards deoxycytidines modified at *N*^4^-position. Mutants *Dm*dNK-V84A and *Dm*dNK-V84G presented phosphorylation efficiencies of up to 98% for deoxycytidines modified with glycine (**3**), alanine (**5**), or isobutyryl- group (**9**), whereas *Dm*dNK-WT phosphorylated the compounds with much lower efficiencies that were between 0% and 31%. The mutants retained the same activity towards *N*^4^-acetyl-2′-deoxycytidine (**8**) as *Dm*dNK-WT and converted the compound into its monophosphate fully.

Replacement of the methionine at position 88 with either alanine (*Dm*dNK-M88A) or glycine (*Dm*dNK-M88G) resulted in a 2- to 6-fold decrease in activity towards all our tested *N*^4^-modified deoxycytidines (**3**–**9**). Phosphorylation efficiencies reached up to 54%, whereas the wild-type enzyme converted the same compounds to their monophosphate forms with efficiencies of up to 100%. In comparison, the double mutant *Dm*dNK-V84A+M88A performed slightly better than the single M88A mutation-carrying variant as conversions of the *N*^4^-modified nucleosides **3**, **5,** and **8** reached 56–100% compared to 1–54%. However, *N*^4^-isobutyryl-2′-deoxycytidine (**9**) was not efficiently phosphorylated by either of the variants carrying an M88 mutation. Variant *Dm*dNK-M88R, which had arginine instead of methionine in position 88, as is natively found in many deoxyguanosine kinases, presented with a complete loss of activity towards all tested *N*^4^-modified nucleosides, as did the double mutant *Dm*dNK-M88R+A110G.

Although alanine is already a small residue, the replacement of A110 to glycine (*Dm*dNK-A110G) led to a significantly higher activity towards the *N*^4^-modified deoxycytidines: phosphorylation efficiencies of **3**, **5**, and **9** increased up to 10-fold compared to *Dm*dNK-WT and reached 30–98%. While replacing A110 with glycine increased the activity of the kinase towards *N*^4^-modified pyrimidine nucleosides, mutation A110D did the opposite. Phosphorylation of substrates **3**, **8**, and **12** by *Dm*dNK-A110D decreased to 0–27%, which were otherwise phosphorylated with efficiencies of 31–100% by *Dm*dNK-WT. In addition, the double mutant *Dm*dNK-V84A+A110D, which presented with a 5-fold decrease in activity towards deoxycytidine compared to *Dm*dNK-WT, was unable to phosphorylate any of the *N*^4^-modified cytidines.

*N*^4^-amino acid-modified deoxycytidines that had a Boc-protecting group (**4**, **6**, and **7**) were not acceptable substrates to the tested *Dm*dNK mutants. Since the modifications are rather large, we believe that they would simply not fit in the active centres of the kinases. In addition, neither of the examined variants with mutations at V84, M88, and A110 positions showed high activity towards the acidic nucleosides **10** and **11**, and *N*^4^-hydroxy- (**12**) and *N*^4^-alkoxy-modified cytidines (**13**, **14**). Phosphorylation efficiencies of the compounds reached up to 24% compared to up to 46% of *Dm*dNK-WT, although in most cases the compounds were not accepted as substrates at all. The modifications are relatively small sized which leads to believe that it is unfavourable interactions and not a lack of space that prevents the compounds from being efficiently phosphorylated.

**Figure 4:**
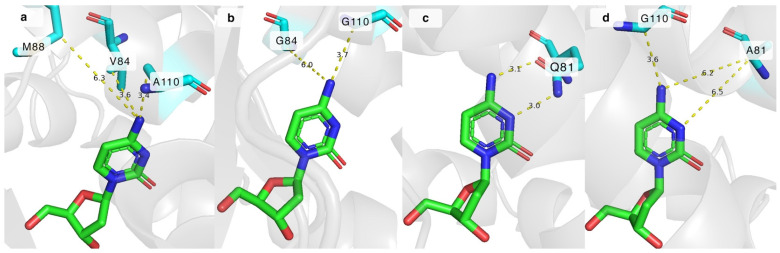
The active site of *Dm*dNK with bound deoxycytidine. (**a**) Distances between *N*^4^ atom of deoxycytidine and V84, M88, and A110 residues of *Dm*dNK-WT. PDB ID: 1j90. (**b**) Predicted distances between *N*^4^ atom of deoxycytidine and residues at positions 84 and 110, where V84 and A110 are replaced by glycine. Model generated by ColabFold v1.5.5d. (**c**) Hydrogen bonds between *N*^3^ and *N*^4^ atoms of deoxycytidine and Q81 residue in *Dm*dNK-WT. PDB ID: 1j90. (**d**) Predicted distances between deoxycytidine and the residues at positions 81 and 110, where Q81 is replaced by alanine, and A110 is replaced by glycine. Model generated by ColabFold v1.5.5.

The impact of the mutations can be explained by analysing the crystal structure of *Dm*dNK-WT with deoxycytidine in its active site (PDB ID: 1j90). As depicted in Figure 4a, residues V84 and A110 are in close proximity to the *N*^4^ atom of deoxycytidine, 3.6 Å and 3.4 Å, respectively. The extra space gained by replacing these residues with smaller amino acids allows the accommodation of bulkier nucleoside analogues (Figure 4b). In addition to the increased physical capacity of the substrate pocket, non-covalent interactions also play a role. The loss of the hydrophobic side chain of valine in the case of *Dm*dNK-V84A or *Dm*dNK-V84G, or methyl in the case of *Dm*dNK-A110G, increases the affinity towards substrates with polar modifications such as *N*^4^-glycinoyl-2′-deoxycytidine (**3**). The methionine at position 88 is 6.3 Å away from the *N*^4^ atom, thus replacing the residue with a more compact group is expected to have less impact on the specificity of *Dm*dNK. As evident from our results, swapping M88 with either a smaller residue such as alanine (*Dm*dNK-M88A) or glycine (*Dm*dNK-M88G) or a positively charged arginine (*Dm*dNK-M88R) had a negative effect on the phosphorylation efficiency—the activity of all variants towards the tested compounds decreased two- to threefold compared to wild-type *Dm*dNK.

To sum up, residues V84 and A110 have significant effects on the phosphorylation efficiency: replacing them with less bulky amino acids creates extra space in the active centre and increases the activity of the kinase towards substrates bearing *N*^4^-modifications. Since residue M88 is rather far away from the *N*^4^-position of cytosine, replacing it with a smaller residue had a negative effect on its activity: a decrease in phosphorylation efficiency was observed.

### 2.4. Significance of the Q81 Residue in the Active Site of DmdNK

Another position of interest was Q81, which forms two hydrogen bonds with the *N*^3^ and *N*^4^ atoms of cytosine and assists in stabilizing the substrate in the active site [25]. Some *Dm*dNK-Q81 mutants have already been described. Replacing the positive charge of glutamine with the negative charge from glutamate was shown to enable phosphorylation of an unnatural pyrimidine nucleoside, 6-amino-5-nitro-3-(1′-β-D-2′-deoxyribofuranosyl)-2(1*H*)-pyridone, that was otherwise repulsed by the wild-type kinase due to the *N*^3^ atom bearing a hydrogen atom and acting as a proton donor instead of an acceptor [35]. In addition, shortening the residue by one carbon atom by replacing glutamine with asparagine caused the enzyme to favour purine nucleosides over pyrimidines [36]. To gain a deeper understanding of the importance of the glutamine residue at position 81, we have created a mutant in which the bulkier residue was replaced with the residue of alanine (*Dm*dNK-Q81A). Our hypothesis was that the removal of the hydrogen bonds between the nucleobase and the residue should allow more flexibility of the nucleobase moiety in the active centre, thus nucleosides with larger modifications would fit more easily. As illustrated in Figure 4c, in the wild-type enzyme the distances between the *N*^3^ and *N*^4^ atoms of the nucleobase and Q81 are 3.0 Å and 3.1 Å. However, according to the in silico ColabFold v1.5.5 model, when Q81 is replaced by alanine (*Dm*dNK-Q81A), the distances increase to over 6.2 Å (Figure 4d) and all interactions are eliminated. As can be seen from Table 1, the mutation alone did not cause the kinase to behave in a drastically different way to the wild-type variant. It retained the same activity towards 2′-deoxycytidine (**1**) and *N*^4^-acetyl-2′-deoxycytidine (**8**) as *Dm*dNK-WT; however, phosphorylation of cytidine (**2**) decreased more than 5-fold. Conversions of the *N*^4^-modified deoxycytidines **3**, **5**, and **9** into the corresponding monophosphates did not differ significantly from *Dm*dNK-WT and reached 0–20%. Just like the wild-type kinase, *Dm*dNK-Q81A was unable to phosphorylate the acidic nucleosides **10** and **11**, and nucleosides bearing the Boc-protecting group (**4**, **6**, and **7**). Even though phosphorylation of *N*^4^-hydroxycytidine (**12**) decreased more than 20-fold, a slight improvement was noticed with *N*^4^-methoxy- (**13**) and *N*^4^-ethoxycytidine (**14**). The compounds were phosphorylated with 4–11% efficiencies, whereas *Dm*dNK-WT reached efficiencies of 0–5%.

The Q81A mutation was paired with the V84G, M88G, or A110G mutations to test whether any cumulative effects were present, but the double mutants *Dm*dNK-Q81A+V84G, *Dm*dNK-Q81A+M88G, and *Dm*dNK-Q81A+A110G did not exhibit improved performance compared to the single-mutation carrying variants *Dm*dNK-V84G, *Dm*dNK-M88G, and *Dm*dNK-A110G. Nevertheless, a double mutant *Dm*dNK-Q81A+A110G showed an improvement in phosphorylating *N*^4^-alkoxy-modified cytidines (**13**, **14**) compared to *Dm*dNK-WT and *Dm*dNK-A110G as conversions of the compounds increased from 0–5% to 30%. Since the compounds were poorly accepted by both *Dm*dNK-WT and all mutant variants, the extra space provided by the A110G mutation, and the additional flexibility of the substrate provided by the removal of the bonds to Q81 may be the reasons (Figure 4d).

### 2.5. Significance of the W57 Residue in the Active Site of DmdNK

**Figure 5:**
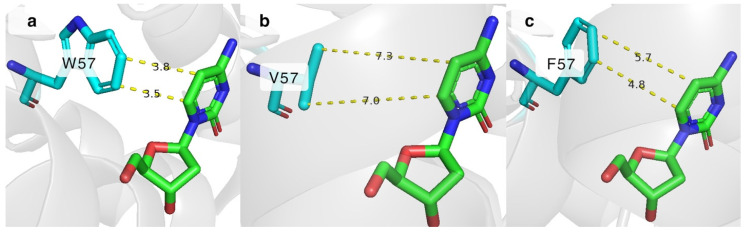
The active site of *Dm*dNK with bound deoxycytidine. (**a**) Stacking interaction between W57 and cytosine atoms. PDB ID: 1j90. (**b**) Predicted distances between C5 and C6 atoms of deoxycytidine and residue at position 57, where W57 is replaced by valine. Model generated by ColabFold v1.5.5. (**c**) Possible interactions between C5 and C6 atoms of deoxycytidine and residue at position 57, where W57 is replaced by phenylalanine. Model generated by ColabFold v1.5.5.

Stacking interactions are important for stabilizing the cytosine nucleobase in place. One of the residues responsible for π-stacking in the active site of *Dm*dNK is W57, which interacts with the C5 and C6 atoms of cytosine as depicted in Figure 5a [25]. It is currently unknown how important this interaction is for the substrate specificity of the enzyme. To gain insight into this issue, we have created two *Dm*dNK mutants in which W57 was replaced by the hydrophobic residues, phenylalanine (*Dm*dNK-W57F) or valine (*Dm*dNK-W57V). The phosphorylation efficiencies of the mutants are provided in Table 1. Compared to *Dm*dNK-WT, the activities of the mutants towards deoxycytidine remained largely unchanged, but the phosphorylation efficiencies of cytidine and all nucleoside analogues tested decreased drastically (from 0–100% to 0–51%). According to the active site models generated by ColabFold v.1.5.5, the distances between cytosine and valine in the case of *Dm*dNK-W57V become too large (over 7.0 Å) for any kind of interaction to occur (Figure 5b). Nonetheless, the distance between the aromatic ring of phenylalanine and cytosine might still allow weak non-covalent interactions (Figure 5c). This explains why the mutant *Dm*dNK-W57F, which had a phenylalanine residue, retained slightly higher activity compared to that of the mutant *Dm*dNK-W57V, which had a valine residue. The results suggest that the stabilizing interaction between the nucleobase and the aromatic system of W57 is crucial for the overall performance of the kinase.

### 2.6. Enhancing BsdCK Substrate Diversity via Single-Site Mutagenesis

**Figure 6:**
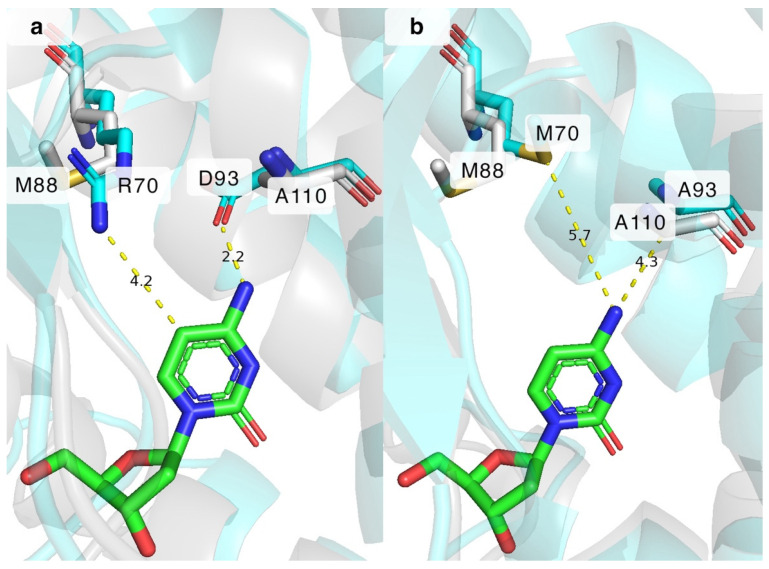
Superimposition of *Bs*dCK (cyan) on *Dm*dNK (grey) with 2′-deoxycytidine (green) in the active centres. (**a**) M88 and A110 residues of *Dm*dNK-WT, and corresponding R70 and D93 residues of *Bs*dCK-WT near the *N*^4^ and C5 positions of 2′-deoxycytidine. (**b**) M88 and A110 residues of *Dm*dNK-WT, and M70 and A93 residues of *Bs*dCK-R70M+D93A mutant variant near the *N*^4^ and C5 positions of 2′-deoxycytidine. Grey residues belong to *Dm*dNK. Cyan residues belong to *Bs*dCK. Yellow dashed lines show predicted distances between the residues of *Bs*dCK and 2′-deoxycytidine. *Dm*dNK PDB ID: 1j90. *Bs*dCK models generated using ColabFold v.1.5.5 software.

*Bs*dCK is known to favour nucleosides of adenine and cytosine, and, as we have discovered in our previous study, it can also phosphorylate various pyrimidine nucleosides bearing small modifications at the nucleobase or sugar [20,23]. While *Bs*dCK demonstrates a relatively broad substrate acceptance, it is still outperformed by the extensively researched *Dm*dNK, which has one of the broadest substrate specificities known for nucleoside kinases [12,19,21,27,35,36,37,38]. Thus, we decided to modify the wild-type *Bs*dCK (*Bs*dCK-WT) to make it more similar to the *Dm*dNK-WT in terms of substrate scope. Although the crystal structure of *Bs*dCK has not been solved, we employed the protein structure prediction software ColabFold v.1.5.5 to determine potentially important residues. The generated model of *Bs*dCK-WT monomer was superimposed on *Dm*dNK-WT, and the residues lining the substrate pockets were compared. Since our goal was to increase the activity of *Bs*dCK towards pyrimidine nucleosides bearing modifications at the *N*^4^ position, we have chosen residues that resided close to this group. As discussed previously, in the active site of *Dm*dNK, residues M88 and A110 are located near the *N*^4^ position of the cytidine nucleobase. In the case of *Bs*dCK, these residues correspond to R70 and D93 (Figure 6a). Single-site mutagenesis was used to generate three mutant variants of *Bs*dCK: two single-mutation carrying variants *Bs*dCK-R70M and *Bs*dCK-D93A, and a double mutant *Bs*dCK-R70M+D93A. The results are provided in Table 2.

The mutant *Bs*dCK-R70M, which had methionine instead of arginine, displayed extremely low activity. Compared to *Bs*dCK-WT, phosphorylation of canonical deoxycytidine (**1**) and cytidine (**2**) decreased from 93–100% to 2–7%. Traces of *N*^4^-glycinoyl-2′-deoxycytidine (**3**) monophosphate were detected in the reaction mixture, although a 5-fold decrease in phosphorylation efficiency was observed when compared to that of the wild-type kinase. Variant *Bs*dCK-R70M exhibited no activity towards the remainder of the *N*^4^-modified nucleosides and will not be further discussed.

The other single mutant, *Bs*dCK-D93A, retained similar activity towards deoxycytidine (**1**) and *N*^4^-acetyl-2′-deoxycytidine (**8**) compared to that of *Bs*dCK-WT, but phosphorylation of cytidine (**2**) still decreased significantly (from 93% to 4%). However, an improvement in the phosphorylation of *N*^4^-modified deoxycytidines was observed. Conversion of *N*^4^-amino acid modified nucleosides **3** and **5** increased from 0–11% to 16–20% compared to that of *Bs*dCK-WT, and deoxycytidine bearing the *N*^4^-isobutyryl modification (**9**) was phosphorylated with 79% efficiency, whereas it was barely accepted by the wild-type kinase (2%).

However, the greatest increase in activity was noticed with the double-mutant *Bs*dCK-R70M+D93A. Similarly to the single-mutation carrying *Bs*dCK-D93A, it retained the same activities as those of *Bs*dCK-WT towards deoxycytidine (**1**) and *N*^4^-acetyl-2′-deoxycytidine (**8**), but a 2-fold decrease in cytidine (**2**) phosphorylation efficiency was observed. The *N*^4^-modified deoxycytidines **3**, **5**, and **9** were converted into their monophosphate forms with high efficiencies, ranging from 37% to 98%. *Bs*dCK-R70M+D93A even outperformed the *Dm*dNK-WT, after which the double mutant was modelled. The mutant exhibited efficiencies comparable to those of the best-performing *Dm*dNK-V84A and *Dm*dNK-V84G variants, even though the corresponding position (F66) in the active site of *Bs*dCK-R70M+D93A remained unchanged.

The introduced mutations of *Bs*dCK were not enough to overcome the spatial limitations of the substrate pocket and/or unfavourable interactions between the residues of the active centre and the substrates: neither of the *Bs*dCK mutants gained the ability to phosphorylate the nucleoside analogues bearing Boc-protecting group (**4**, **6** and **7**), or the acidic nucleosides **10** and **11**. A decrease in activity towards *N*^4^-hydroxy- (**12**) and *N*^4^-alkoxy-modified nucleosides (**13** and **14**) was observed, as well. The best-performing double mutant *Bs*dCK-R70M+D93A displayed a 2- to 7-fold decline in phosphorylation efficiency when compared to that of *Bs*dCK-WT (conversion went from 1–18% to 0–9%).

The increased activity towards *N*^4^-modified deoxycytidines **3**, **5**, and **9** can be partially explained by the extra space gained by replacing the bulkier aspartate with the smaller alanine at position 93. As depicted in Figure 6a, the aspartate residue in the *Bs*dCK-WT is expected to be located 2.2 Å from the cytosine nucleobase and form hydrogen bonds with its *N*^4^ atom. This explains why cytosine moiety-bearing nucleosides are such excellent substrates to the wild-type enzyme. The aspartate, however, is not so great for the *N*^4^-modified deoxycytidines. Both the proximity and the negative charge of the aspartate residue prevent many nucleoside analogues from fitting into the active site of the enzyme. The issues are remedied by replacing the residue with alanine as it is found in *Dm*dNK-WT. As can be seen from Figure 6b, replacing the D93 residue with alanine lengthens the distance from 2.2 Å to 4.3 Å, which in turn allows for the accommodation of nucleoside analogues that are modified at the *N*^4^-position. In the case of arginine at position 70, the residue is expected to be located 4.2 Å from the C5 carbon (Figure 6a) which is far enough to accommodate canonical pyrimidine nucleosides in the active site. However, the positively charged tail of arginine prevents nucleosides with certain modifications at positions *N*^4^ or C5 from entering the active site, both by spatial limitation and by the positive charge. This explains why, as was discovered in our previous study, thymidine is a poor substrate for the *Bs*dCK-WT. Replacing R70 with a hydrophobic methionine, as is natively found in *Dm*dNK-WT, provides additional space in the active centre (Figure 6b), as well as removes the positive charge provided by arginine. In conclusion, mimicking the active site of *Dm*dNK by replacing both R70 with methionine and D93 with alanine has proven to be an excellent way to increase the activity of *Bs*dCK towards *N*^4^-modified cytosine nucleosides.

### 2.7. Differences in Phosphorylating N^4^-Amino Acid Modified Nucleosides Bearing Amide and Carboxyl Functional Groups

**Figure 7:**
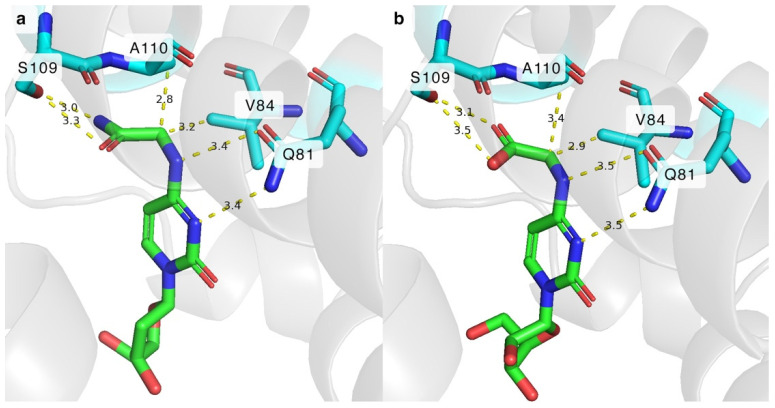
(**a**) Molecular docking of *N*^4^-glycinoyl-2′-deoxycytidine in the active centre of *Dm*dNK-WT. (**b**) Molecular docking of *N*-[1-(β-D-ribofuranosyl)-2-oxo-4-pyrimidinyl]-glycine in the active centre of *Dm*dNK-WT. PDB ID: 1j90. Molecular docking performed by DiffDock software (https://huggingface.co/spaces/reginabarzilaygroup/DiffDock-Web; accessed on 6 August 2024).

As it became apparent that the primary amide group bearing nucleosides *N*^4^-glycinoyl-2′-deoxycytidine (**3**) and *N*^4^-alaninoyl-2′-deoxycytidine (**5**) were successfully phosphorylated by certain *Dm*dNK and *Bs*dCK mutants, we decided to test whether analogues with carboxyl groups instead of amide functional groups were suitable substrates for the mutants, as well. Hence, two acidic cytidines bearing glycine and alanine residues (**10**, **11**) were synthesized. To our surprise, none of the *Dm*dNK or *Bs*dCK variants were capable of phosphorylating the compounds. Since the amide and carboxyl groups are similar in size, we hypothesized that the issue was not a lack of space, but rather unfavourable interactions between the substrate and the residues of the enzymes’ active sites. To gain insight into how the *N*^4^-modified nucleoside might bind to the enzyme’s active centre, *Dm*dNK-WT was docked with its substrate *N*^4^-glycinoyl-2′-deoxycytidine (**3**) and a non-accepted *N*^4^-glycine-modified acidic nucleoside (**10**). As depicted in Figure 7a,b, both compounds form very similar interactions with neighbouring residues Q81, V84, and A110. In addition, the amide and carboxyl groups of the nucleosides form interactions with the residue S109, which we expect to be the determining factor in whether the *N*^4^-amino acid-modified nucleoside is phosphorylated or not. The reason why **3** was efficiently phosphorylated by certain *Dm*dNK mutants was favourable interactions. The amine of the amide group forms hydrogen bonds with the hydroxyl group of the serine 109 residue. On the other hand, **10** was not a suitable substrate for any of the kinase variants tested, and we believe that the same serine residue is the culprit. Under the conditions we tested (pH 7.5), the carboxyl group of glycine in the acidic nucleoside **10** is expected to be deprotonated and carry a negative charge, creating an unfavourable repulsive interaction between the nucleoside and the S109 residue [39]. In the given in silico models, the carboxyl group bearing nucleoside can be seen to be shifted slightly further away from S109, and closer to V84, compared to the nucleoside bearing the *N*^4^-amide functional group. We believe that the repulsive force prevents the acidic nucleoside from entering the active sites of the kinases and it is the reason why one compound is accepted as a substrate and the other one is not. To prove whether this theory is correct, additional research should be performed.

## 3. Materials and Methods

### 3.1. General Information

Chemicals and solvents were purchased from Sigma–Aldrich (Darmstadt, Germany), Alfa Aesar (Kandel, Germany), Honeywell (Machelen, Belgium), Fluka (Buchs, Switzerland), Merck (Vilnius, Lithuania) and GE Healthcare (Freiburg, Germany), and were of analytical grade or higher. A full list of nucleosides, utilized for the study, is provided in Supplementary Data. Thin-layer chromatography (TLC) was carried out on 25 TLC aluminium sheets coated with silica gel 60 F254 (Merck, Vilnius, Lithuania). Ion exchange chromatography was carried out on Dowex 1 × 8 formate form (Sigma–Aldrich, Darmstadt, Germany). Flash chromatography was performed using Buchi Pure C-815 Flash system, equipped with a diode array detector (Buchi AG, Flawil, Switzerland). NMR spectra were recorded in DMSO-d_6_ on a Bruker Ascend 400 ^1^H NMR—400 MHz, ^13^C NMR—101 MHz, ^31^P NMR—202 MHz (Bruker BioSpin, Rheinstetten, Germany). Chemical shifts are reported in ppm relative to the solvent resonance signal as an internal standard. UV spectra were recorded on a Lambda 25 Perkin Elmer UV/VIS spectrometer (PerkinElmer, Beaconsfield, UK). HPLC-MS analyses were performed using a high-performance liquid chromatography system, equipped with a photodiode array detector (SPD-M20A) and a mass spectrometer (LCMS-2020, Shimadzu, Kyoto, Japan) equipped with an ESI source. The chromatographic separation was conducted using a YMC Pack Pro column, 3 × 150 mm (YMC, Kyoto, Japan) at 40 °C and a mobile phase that consisted of 0.1% formic acid water solution (solvent A), and acetonitrile (solvent B). Mass spectrometry data was acquired in both positive and negative ionization mode and analysed using the LabSolutions LCMS software (version 5.82 SP1). Protein structures were visualized using the PyMOL Molecular Graphics System, Version 3.0 Schrödinger, LLC. Protein structure models were generated by ColabFold v1.5.5 software [40]. Molecular docking was performed utilizing DiffDock software (https://huggingface.co/spaces/reginabarzilaygroup/DiffDock-Web; accessed on 6 August 2024) [41].

### 3.2. Synthesis of N-[1-(β-D-Ribofuranosyl)-2-oxo-4-pyrimidinyl]-amino Acids

4-Thiouridine (3.85 mmol) was dissolved in 8 mL *N*,*N*-dimethylformamide, 5.8 mmol 2,4-dinitrofluorobenzene was added and the reaction mixture was stirred for 1 h at 50 °C temperature. The reaction was monitored by TLC analysis. After the reaction was completed, the reaction mixture was concentrated using a rotary evaporator and the residue was purified by column chromatography (silica gel, chloroform/methanol mixture, 100:0 → 93:7).

Appropriate amino acid (10 mmol) was dissolved in 10 mL 50 mM potassium phosphate buffer (pH 7.5) and activated thioether (220 mg, 5 mmol) was added to the reaction mixture which was then stirred for 3 days at 40 °C. The reaction was monitored with TLC analysis. After the reaction was completed, the reaction mixture was washed with chloroform to eliminate residual activated thioether.

*N*-[1-(β-D-ribofuranosyl)-2-oxo-4-pyrimidinyl]-glycine was purified by ion-exchange chromatography using Dowex I column (formate, 2.5 × 12 cm), washed with water, and eluted with 0.1 M formic acid. The fractions containing the product were combined, washed with ether, and concentrated to obtain a white solid compound.

*N*-[1-(β-D-ribofuranosyl)-2-oxo-4-pyrimidinyl]-L-alanine was purified by flash chromatography. Firstly, the product was separated using FlashPure Select C18 30µm spherical 20 g column (water/methanol mixture, 95:5 → 0:100). The fractions containing the product were combined and concentrated to 1 mL using a rotary evaporator. To remove residue amino acid, a second purification was performed using FlashPure EcoFlex Amino 50 µm spherical column (acetonitrile/10 mM ammonium carbonate (pH 8.0) buffer, 95:5 → 60:40).

Synthesized nucleosides were characterized by NMR spectroscopy and HPLC-MS analysis.

### 3.3. Synthesis of N^4^-Hydroxy- and N^4^-Alkoxycytidines

Cytidine (8.23 mmol) was dissolved in 0.4 mL water, 9.05 mmol of corresponding amine hydrochloride salt (hydroxylamine, methoxyamine, or ethoxyamine) was added and the reaction mixture was heated at 70 °C temperature. The reaction was monitored with TLC analysis. After the reaction was completed, the reaction mixture was concentrated using a rotary evaporator and the residue was purified by column chromatography (silica gel, chloroform/methanol mixture, 100:0 → 80:20). The products were obtained in 56–76% yields. Synthesized nucleosides were characterized by NMR spectroscopy and HPLC-MS analysis.

### 3.4. Bacterial Strains, Plasmids, and Reagents

Construction of plasmids, that contained *Dm*dNK-WT and *Bs*dCK-WT genes, has been previously described [23]. *Escherichia coli* DH5α was used for plasmid amplification. *Escherichia coli* BL21 (DE3) and KRX strains were employed for the expression of target genes. All three strains were purchased from Novagen (Darmstadt, Germany). Thermo Scientific aLICator LIC cloning and expression kit was purchased from ThermoFisher Scientific (Vilnius, Lithuania). Phusion Plus PCR Master Mix, utilized for gene amplification, was purchased from ThermoFisher Scientific (Vilnius, Lithuania). Restriction enzyme DpnI (10 U/μL) was purchased from ThermoFisher Scientific (Vilnius, Lithuania).

### 3.5. Site-Directed Mutagenesis

The mutant variants of *D. melanogaster* dNK and *B. subtilis* dCK were created using site-directed mutagenesis. Plasmids pLATE31-dNK and pLATE31-dCK were used as starting templates [23]. All templates and primers utilized in the construction of the mutants are listed in Table S1. The gene syntheses were performed by PCR using Phusion Plus polymerase. *Dm*dNK variants V84A, V84A+A110D, V84A+M88A, M88A, M88R, M88R+A110D, and A110D, and *Bs*dCK variants R70M, R70M+D93M, and D93M were synthesized conventionally, using a forward and a reverse primer, both carrying the same mutation. *Dm*dNK variants W57F, W57V, Q81A, Q81A+V84G, Q81A+M88G, Q81A+A110G, V84G, M88G, and A110G were created using a single-primer site-directed mutagenesis protocol as described by Huang and Zhang [42]. PCR mixtures were treated with 1 µL of DpnI to remove the template DNA. The amplified genes were purified from 1% agarose gel and cloned into pLATE31 plasmid according to the manufacturer’s protocol. The resulting plasmids were chemically transformed into competent *E. coli* DH5α cells. Transformants were selected on LB agar plates (0.1 mg/mL ampicillin). The plasmids were isolated and sequenced to confirm that the desired mutations were present. The plasmids with correct sequences were named pLATE31-dNK-W57F, pLATE31-dNK-W57V, pLATE31-dNK-Q81A, pLATE31-dNK-Q81A+V84G, pLATE31-dNK-Q81A+M88G, pLATE31-dNK-Q81A+A110G, pLATE31-dNK-V84A, pLATE31-dNK-V84G, pLATE31-dNK-V84A+M88A, pLATE31-dNK-V84A+A110D, pLATE31-dNK-M88A, pLATE31-dNK-M88G, pLATE31-dNK-M88R, pLATE31-dNK-M88R+A110D, pLATE31-dNK-A110D, pLATE31-dNK-A110G, pLATE31-dCK-R70M, pLATE31-dCK-R70M+D93M, and pLATE31-dCK-D93M.

### 3.6. Expression of Target Genes and Purification of Proteins

The recombinant plasmids, except for *Dm*dNK-Q81A+V84G, *Dm*dNK-Q81A+M88G, *Dm*dNK-Q81A+A110G, *Dm*dNK-V84G, *Dm*dNK-M88G, and *Dm*dNK-A110G, for which *E. coli* KRX strain was used, were transformed into *E. coli* BL21 (DE3) cells by electroporation. Transformants, selected on 0.1 mg/mL ampicillin LB agar plates, were inoculated into 200 mL of LB medium containing 0.1 mg/mL ampicillin and incubated at 37 °C and a shaking speed of 180 rpm for 2–4 h. At an OD600 of 0.6, gene expression was induced with 1 mM of IPTG for 3 h. In the case of *E. coli* KRX transformants, at an OD600 of 0.6 the temperature was decreased to 20 °C and gene expression was induced with 0.5 mM of IPTG for 20 h. Cells were harvested by centrifugation at 4000× *g* at 4 °C for 10 min. The supernatant was discarded, and the pellet was suspended in 6 mL of 50 mM potassium phosphate buffer (pH 7.5). Cells were lysed by sonication (5 min total time; 2 s disruption, 8 s cooling; 22 kHz at 40% amplitude) on ice. Cell-free fractions were recovered by centrifugation at 16,000× *g* at 4 °C for 10 min. The obtained lysate was loaded onto 1 mL Ni^2+^ HiTrap chelating HP column equilibrated with 50 mM potassium phosphate buffer (pH 7.5). The enzymes were eluted using 50 mM potassium phosphate buffer containing 0.5 M imidazole (pH 7.5) gradient from 0% to 100%, at a flow rate of 0.7 mL/min. Protein purification was monitored by 14% sodium dodecyl sulphate-polyacrylamide gel electrophoresis (SDS-PAGE). Fractions containing target protein were pooled, placed into dialysis bags, and dialyzed overnight at 4 °C in 1 L of 50 mM potassium phosphate buffer (pH 7.5). The precipitated proteins were removed by centrifugation at 16,000× *g* at 4 °C for 10 min. Concentrations of the purified enzymes were determined to be between 0.04 and 2.4 mg/mL. Solutions of the purified enzymes were stored at −20 °C until further use.

### 3.7. Substrate Specificity Assay

The substrate specificity assays were performed in 50 µL reaction mixtures that contained 10 mM nucleoside, 15 mM GTP, 15 mM MgCl_2_, and 0.69 nmol/mL *Dm*dNK or 2.69 nmol/mL *Bs*dCK in 50 mM potassium phosphate buffer (pH 7.5). The phosphorylation reactions were carried out at 37 °C and a mixing speed of 300 rpm for 24 h. After the reactions were complete, 50 µL of acetonitrile was added. The mixtures were centrifuged at 16,000× *g* at 4 °C for 10 min, supernatants were analysed using HPLC-MS. The substrate specificities of the kinases were determined based on the formation of corresponding nucleoside 5′-monophosphates. The phosphorylation efficiencies were calculated from HPLC-MS chromatogram areas (data provided in Supplementary Materials). Substrates utilized in the study are provided in Figure 3.

## 4. Conclusions

In this study, single-site mutagenesis was applied to two nucleoside kinases *Dm*dNK and *Bs*dCK in order to expand their substrate scope. It was discovered that modifying the substrate pocket of *Dm*dNK by replacing residues V84 with alanine or glycine, and A110 with glycine had the most prominent effects on substrate selectivity. The kinase gained the ability to phosphorylate *N*^4^-modified cytidine nucleosides, such as *N*^4^-glycinoyl-2′-deoxycytidine and *N*^4^-isobutyryl-2′-deoxycytidine, that were otherwise phosphorylated poorly or not at all by the wild-type variant.

Mimicking the active site of *Dm*dNK by replacing residues R70 and D93 with methionine and alanine, respectively, in *Bs*dCK was found to widen the range of its suitable substrates. The double mutant acquired the ability to phosphorylate cytidine analogues, such as *N*^4^-alaninoyl-2′-deoxycytidine and *N*^4^-isobutyryl-2′-deoxycytidine, that were too bulky for *Bs*dCK-WT.

This study proves that manipulating the active sites of nucleoside kinases is a viable option to expand the variety of accepted substrates. With the help of in silico modelling, important residues in the active centres can be recognized and, consequentially, modified to fit a target nucleoside. The strategy can be employed for enzymatic nucleoside 5′-triphosphate synthesis, where the first phosphorylation step is usually performed by nucleoside kinases [17,23,24,43,44]. The following steps are performed using nucleoside monophosphate and nucleoside diphosphate kinases. Similar to nucleoside kinases, nucleoside monophosphate kinases are known to be substrate-specific. For example, viral thymidylate kinases HSV-1 TK and VZV TK phosphorylate 2′-deoxythymidine, dTMP and dUMP, while mammalian UMP-CMP kinase accepts CMP, dCMP, UMP, dUMP, AMP, and dAMP as substrates [11,45]. Nucleoside diphosphate kinases, on the other hand, possess a wide-substrate cleft and can phosphorylate an abundance of substrates [11,46,47]. In addition to nucleotide synthesis, nucleoside kinases, such as *Dm*dNK or HSV1-TK, are often utilized in suicide gene therapy where they activate nucleoside prodrugs that are targeted towards tumour cells [9,48,49,50]. Modifying the kinases can be an effective strategy for improving their activity towards target nucleosides and, in turn, increasing the efficacy of cancer treatment [34,51]. Furthermore, the kinases could be utilized in the field of nucleic acid labelling. For example, *Hs*dCK and *Hs*UCK2 have been successfully applied in cell-specific metabolic labelling of RNA, where the kinases phosphorylated 2′-azidocytidine and 2′-azidouridine, respectively, into their monophosphate forms, which were then subsequently phosphorylated into NTPs by cellular NMP and NDP kinases. Incorporation of these NTPs by RNA polymerases allowed for effective RNA probing with low cytotoxicity [4,5].

## Figures and Tables

**Table 1:** Phosphorylation efficiencies of pyrimidine nucleosides by *Dm*dNK wild-type and mutant variants. Number is the phosphorylation efficiency (%) calculated using HPLC-MS chromatogram areas. Cells are coloured based on mutant performance in comparison to the wild-type kinase with the particular substrate (a margin of error of ±3 was applied). Rose—phosphorylation efficiency decreased ≥2-fold compared to *Dm*dNK-WT; orange—phosphorylation efficiency decreased ≥1.5-fold but <2-fold compared to *Dm*dNK-WT; lime—phosphorylation efficiency increased ≥1.5-fold but <2-fold compared to *Dm*dNK-WT; green—phosphorylation efficiency increased ≥2-fold compared to *Dm*dNK-WT; white—phosphorylation efficiency was similar to *Dm*dNK-WT.

	Substrate	1	2	3	4	5	6	7	8	9	10	11	12	13	14
*Dm*dNK															
WT		98	78	31	0	3	0	0	100	0	0	0	46	5	0
W57F		89	51	1	0	0	0	0	27	1	0	0	6	0	0
W57V		89	6	0	0	0	0	0	4	0	0	0	0	0	0
Q81A		88	14	20	0	0	0	0	100	9	0	0	2	11	4
Q81A+V84G		92	2	82	0	31	0	0	88	61	0	0	0	4	3
Q81A+M88G		94	4	7	0	1	0	0	93	3	0	0	0	6	1
Q81A+A110G		93	13	98	0	55	0	0	100	87	0	0	11	30	30
V84A		97	75	95	0	75	0	0	100	68	0	0	24	8	2
V84A+M88A		98	71	60	0	56	0	0	100	0	0	0	1	3	2
V84A+A110D		22	1	0	0	0	0	0	0	0	0	0	0	0	0
V84G		88	41	98	0	84	0	0	100	94	0	0	4	6	2
M88A		79	47	14	0	1	0	0	54	4	0	0	9	0	0
M88G		87	30	5	0	1	0	0	29	2	0	0	6	0	0
M88R		34	5	1	0	0	0	0	0	0	0	0	0	0	0
M88R+A110G		91	4	0	0	0	0	0	0	0	0	0	0	0	0
A110D		96	10	11	0	0	0	0	27	0	0	0	0	0	0
A110G		89	65	98	0	33	0	0	100	30	0	0	8	4	0

**Table 2:** Phosphorylation efficiencies of pyrimidine nucleosides by *Bs*dCK wild-type and mutant variants. Number is the phosphorylation efficiency (%) calculated using HPLC-MS chromatogram areas. Cells are coloured based on mutant performance in comparison to the wild-type kinase with the particular substrate (a margin of error of ±3 was applied). Rose—phosphorylation efficiency decreased ≥2-fold compared to *Bs*dCK-WT; orange—phosphorylation efficiency decreased ≥1.5-fold but <2-fold compared to *Bs*dCK-WT; lime—phosphorylation efficiency increased ≥1.5-fold but <2-fold compared to *Bs*dCK-WT; green—phosphorylation efficiency increased ≥2-fold compared to *Bs*dCK-WT; white—phosphorylation efficiency was similar to *Bs*dCK-WT.

	Substrate	1	2	3	4	5	6	7	8	9	10	11	12	13	14
*Bs*dCK															
WT		100	93	11	0	0	0	0	95	2	0	0	18	1	7
R70M		7	2	2	0	0	0	0	0	0	0	0	0	0	0
R70M+D93A		100	55	98	0	37	0	0	99	98	0	0	9	0	1
D93A		100	5	20	0	16	0	0	97	79	0	0	2	0	0

## Data Availability

The original contributions presented in the study are included in the article, further inquiries can be directed to the corresponding author.

## Supplementary Materials

The following supporting information can be downloaded at: https://www.mdpi.com/article/10.3390/molecules29163767/s1, a list of used reagents, ^1^H-NMR (400 MHz), ^13^C-NMR (101 MHz) and MS spectra of compounds. Table S1: PCR primers used for site-directed mutagenesis of *Dm*dNK and *Bs*dCK. Figure S1: HPLC chromatogram of *Dm*dNK-V84A catalysed *N*^4^-isobutyryl-deoxycytidine phosphorylation reaction. Table S2: HPLC data of *Dm*dNK catalysed reactions. Table S3: HPLC data of *Bs*dCK catalysed reactions.
