# Supplementary material for: Exploring the Mutated Kinases for Chemoenzymatic Synthesis of N4-Modified Cytidine Monophosphates
Source: Molecules. 2024 Aug 9;29(16):3767. doi: 10.3390/molecules29163767 (PMC11357392; doi:10.3390/molecules29163767)
Supplement: Supplementary file 1 [file molecules-29-03767-s001.zip › molecules-3128395-supplementary.pdf]

Supplementary Material

Exploring the Mutated Kinases for Chemoenzymatic Synthesis of N^4-Modified Cytidine Monophosphates

Martyna Koplūnaitė *,†, Kamilė Butkutė †, Jonita Stankevičiūtė and Rolandas Meškys *

Department of Molecular Microbiology and Biotechnology, Institute of Biochemistry, Life Sciences Center, Vilnius University, Sauletekio Av. 7, LT-10257 Vilnius, Lithuania; kamile.butkute@bchi.stud.vu.lt (K.B.); jonita.stankeviciute@bchi.vu.lt (J.S.)

* Correspondence: martyna.koplunaite@gmc.vu.lt (M.K.); rolandas.meskys@bchi.vu.lt (R.M.)

† The authors contributed equally to this work.

Reagents

2'-Deoxycytidine (**1**) was purchased from Carbosynth (Compton, UK). Cytidine (**2**) was purchased from Acros Organics (Geel, Belgium). *N*4-Acetyl-2'-deoxycytidine (**8**) and *N*4-isobutyryl-2'-deoxycytidine (**9**) were purchased from Combi-Blocks (San Diego, CA, USA). 4-Thiouridine was purchased from Biosynth (Bratislava, Slovakia). 2,4-Dinitrofluorobenzene was purchased from ThermoFisher Scientific (Vilnius, Lithuania). 2-((1-((2*R*,4*S*,5*R*)-4-Hydroxy-5-(hydroxymethyl)tetrahydrofuran-2-yl)-2-oxo-1,2-dihydropyrimidin-4-yl)amino)acetamide (**3**), *tert*-butyl (2-((1-((2*R*,4*S*,5*R*)-4-hydroxy-5-(hydroxymethyl)tetrahydrofuran-2-yl)-2-oxo-1,2-dihydropyrimidin-4-yl)amino)-2-oxoethyl)carbamate (**4**), (*S*)-2-((1-((2*R*,4*S*,5*R*)-4-hydroxy-5-(hydroxymethyl)tetrahydrofuran-2-yl)-2-oxo-1,2-dihydropyrimidin-4-yl)amino)propenamide (**5**), *tert*-butyl ((*S*)-1-((1-((2*R*,4*S*,5*R*)-4-hydroxy-5-(hydroxymethyl)tetrahydrofuran-2-yl)-2-oxo-1,2-dihydropyrimidin-4-yl)amino)-1-oxopropan-2-yl)carbamate (**6**), and *tert*-butyl (1-((1-((2*R*,4*S*,5*R*)-4-hydroxy-5-(hydroxymethyl)tetrahydrofuran-2-yl)-2-oxo-1,2-dihydropyrimidin-4-yl)amino)-4-methyl-1-oxopentan-2-yl)carbamate (**7**), were synthesized as described previously [1].

Table S1. PCR primers used for site-directed mutagenesis of *Dm*dNK and *Bs*dCK.

Variant	Template	Primer name	Primer Sequence 5' to 3'
*Dm*dNK-W57F	pLATE31-dNK	dNK-W57F-F	GAGCCGGTTGAAAAAttcCGTAATGTTAATGGTG
*Dm*dNK-W57V	pLATE31-dNK	dNK-W57V-F	GAGCCGGTTGAAAAAgtgCGTAATGTTAATGGTG
*Dm*dNK-Q81A	pLATE31-dNK	dNK-Q81A-F	GCAATGCCGTTTgcgAGCTATGTTACCCTGAC
*Dm*dNK-Q81A+V84G	pLATE31-dNK-Q81A	dNK-Q81A+V84G-F	GTTTgcgAGCTATGgTACCCTGACCATGCTGCAG
*Dm*dNK-Q81A+M88G	pLATE31-dNK-Q81A	dNK-M88G-F	GTTACCCTGACCgggCTGCAGAGCCATACCGCACCGAC
*Dm*dNK-Q81A+A110G	pLATE31-dNK-Q81A	dNK-A110G-F	GAACGCAGCATCTTCAGCggaCGTTATTGTTTTG
*Dm*dNK-V84A	pLATE31-dNK	dNK-V84A-F	CAGAGCTATgcgACCCTGACCATGCTGCAG
		dNK-V84A-R	GTCAGGGTcgcATAGCTCTGAAACGGCATTG

*Dm*dNK-V84G	pLATE31-dNK	dNK-V84G-F	GTTTCAGAGCTATggtACCCTGACCATGCTGCAG
*Dm*dNK-V84A+M88A	pLATE31-dNK-V84A	dNK-V84A+M88A-F	GCTATgcgACCCTGACCgcgCTGCAGAGCCATACCGCAC
		dNK-V84A+M88A-R	cgcGGTCAGGGTcgcATAGCTCTGAAACGGCATTGC
*Dm*dNK-V84A+A110D	pLATE31-dNK-V84A	dNK-A110D-F	CATCTTCAGCgatCGTTATTGTTTTGTTGAAAATATG
		dNK-A110D-R	CAAAACAATAACGatcGCTGAAGATGCTGCGTTC
*Dm*dNK-M88A	pLATE31-dNK	dNK-M88A-F	gcgCTGCAGAGCCATACCGCACCGACCAACAAAAAACT
		dNK-M88A-R	ATAGCTCTGAAACGGCATTGCCCATTTTTTCGGATC
*Dm*dNK-M88G	pLATE31-dNK	dNK-M88G-F	GTTACCCTGACCgggCTGCAGAGCCATACCGCACCGAC
*Dm*dNK-M88R	pLATE31-dNK	dNK-M88R-F	GTTACCCTGACCaggCTGCAGAGCCATACCGCAC
		dNK-M88R-R	GCTCTGCAGcctGGTCAGGGTAACATAGC
*Dm*dNK-M88R-A110D	pLATE31-dNK-M88R	dNK-A110D-F	CATCTTCAGCgatCGTTATTGTTTTGTTGAAAATATG
		dNK-A110D-R	CAAAACAATAACGatcGCTGAAGATGCTGCGTTC
*Dm*dNK-A110D	pLATE31-dNK	dNK-A110D-F	CATCTTCAGCgatCGTTATTGTTTTGTTGAAAATATG
		dNK-A110D-R	CAAAACAATAACGatcGCTGAAGATGCTGCGTTC
*Dm*dNK-A110G	pLATE31-dNK	dNK-A110G-F	GAACGCAGCATCTTCAGCggaCGTTATTGTTTTG
*Bs*dCK-R70M	pLATE31-dCK	dCK-R70M-F	atgTTCAAAGAACAGAAAACAATTTTTGAAGC
		dCK-R70M-R	AAGTAAATTTGAAGGTGAAAGCTCCAACG
*Bs*dCK-R70M+D93M	pLATE31-dCK-R70M	dCK-D93M-F	GATTTATGAAgcgACAGGAATTTTCGCAAAAATG
		dCK-D93M-R	GAAAATTCCTGTcgcTTCATAAATCGAACGATC
*Bs*dCK-D93M	pLATE31-dCK	dCK-D93M-F	GATTTATGAAgcgACAGGAATTTTCGCAAAAATG
		dCK-D93M-R	GAAAATTCCTGTcgcTTCATAAATCGAACGATC

Data on synthesized N^4-modified nucleosides

1-(3,4-Dihydroxy-5-(hydroxymethyl)oxolan-2-yl)-4-((2,4-dinitrophenyl)sulfanyl) pyrimidin-2(1H)-one

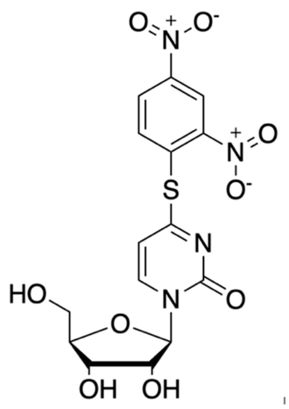

Yield 390 mg (24%), yellow solid.
MS (ESI$^+$): *m/z* 427 [M+H]$^+$, 425 [M-H]$^-$. UV (CH_3OH) λ_{max} 244, 311 nm.
^{1}H NMR (DMSO-d_6, 400 MHz): δ = 3.60 (dd, *J* = 12.4, 2.5 Hz, 1H, CH_2), 3.76 (dd, *J* = 12.2, 2. 5 Hz, 1H, CH_2), 3.94 (q, *J* = 6.0, 5.5 Hz, 1H, CH), 3.94-3.99 (m, 2H, 2CH), 5.07 (s, 1H, OH), 5.25 (s, 1H, OH), 5.59 (s, 1H, OH), 5.68 (d, *J* = 1.9 Hz, 1H, CH), 6.66 (d, *J* = 7.1 Hz, 1H, CH=CH), 8.22 (d, *J* = 8.7 Hz, 1H, CH=CH), 8.51 (d, *J* = 7.1 Hz, 1H), 8.56 (dd, *J* = 8.7, 2.5 Hz, 1H, CH=CH), 8.89 (d, *J* = 2.5 Hz, 1H, CH).
^{13}C NMR (DMSO-d_6, 101 MHz): δ = 59.89, 68.68, 74.98, 84.58, 91.07, 102.93, 120.92, 127.58, 130.86, 138.91, 144.44, 148.23, 151.21, 152.88, 173.17.

***N*-[1-(β-D-Ribofuranosyl)-2-oxo-4-pyrimidinyl]-glycine (10)**

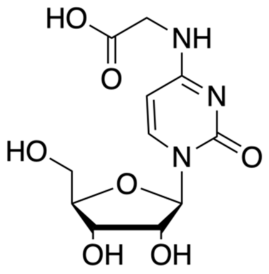

Yield 20 mg (13 %), white solid.
MS (ESI$^+$): *m/z* 302 [M+H]$^+$, 300 [M-H]$^-$. UV (H_2O) λ_{max} 280 nm.
^{1}H NMR (D_2O, 400 MHz): δ = 3.73 (dt, *J* = 12.9, 3.4 Hz, 1H, CH_2), 3.87 (ddd, *J* = 12.9, 7.8, 2.7 Hz, 1H, CH_2), 4.04-4.10 (m, 1H, CH), 4.10-4.17 (m, 3H, $2CH_2$ and CH), 4.26 (ddd, *J* = 8.8, 5.0, 3.4 Hz, 1H, CH), 5.81 (d, *J* = 3.5 Hz, 1H, CH), 6.18 (d, *J* = 7.9 Hz, 1H, CH=CH), 7.93 (d, *J* = 7.9 Hz, 1H, CH=CH), 8.12-8.23 (m, 1H, NH).
^{13}C NMR (D_2O, 101 MHz): δ = 44.13, 60.30, 68.93, 74.03, 90.41, 95.97, 142.04, 145.53, 158.97, 165.97.

***N*-[1-(β-D-Ribofuranosyl)-2-oxo-4-pyrimidinyl]-L-alanine (11)**

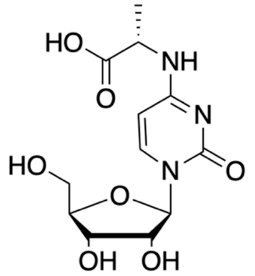

Yield 21 mg (13 %), white solid.
MS (ESI$^+$): *m/z* 316 [M+H]$^+$, 314 [M-H]$^-$. UV (H_2O) λ_{max} 280 nm.
^{1}H NMR (D_2O, 400 MHz): δ = 1.26-1.37 (m, 3H, CH_3), 3.64 (dd, *J* = 12.8, 4.3 Hz, 1H, CH_2), 3.72-3.82 (m, 1H, CH_2), 3.97 (tt, *J* = 6.3, 3.2 Hz, 1H, CH), 4.01-4.09 (m, 1H, CH), 4.16 (dt, *J* = 11.5, 4.3 Hz, 1H, CH), 4.23 (q, *J* = 7.2 Hz, 1H, CH), 5.72 (d, *J* = 3.8 Hz, 1H, CH), 5.96 (d, *J* = 7.8 Hz, 1H, CH=CH), 7.71 (d, *J* = 7.8 Hz, 1H, CH=CH), 8.03 (d, *J* = 8.0 Hz, 1H, NH).
^{13}C NMR (D_2O, 101 MHz): δ = 14.65, 49.59, 58.27, 66.87, 71.62, 81.59, 87.86, 94.29, 138.55, 151.94, 158.50, 175.66.

N^4-Hydroxycytidine (12)

Yield 162 mg (76%), white solid.
MS (ESI$^+$): *m/z* 260 [M+H]$^+$, 258 [M-H]$^-$. UV ($CHCl_3$) λ_{max} 232; 275 nm.
^{1}H NMR (DMSO-d6): δ = 3.53 (dtd, *J* = 15.9; 11.8; 3.8 Hz, 2H 2CH), 3.78 (q, *J* = 3.4 Hz, 1H, CH), 3.93 (q, *J* = 3.8 Hz, 1H, CH), 3.98 (q, *J* = 5.5 Hz, 1H, CH), 4.95–5,07 (m, 2H, CH and OH), 5.24 (d, *J* = 5.9 Hz, 1H, OH), 5.57 (d, *J* = 8.2 Hz, 1H, CH=CH), 5.74 (d, *J* = 6.2 Hz, 1H, OH), 7.05 (d, *J* = 8.2 Hz, 1H, CH=CH), 9.47 (s, 1H, OH), 9.98 (s, 1H, NH).
^{13}C NMR (DMSO-d6): δ = 61.83, 70.78, 72.87, 84.99, 87.21, 98.91, 130.57, 143.95, 150.05.

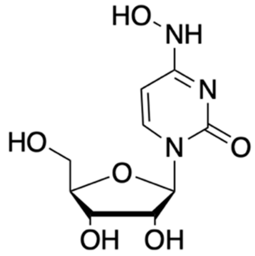

N^4-Methoxycytidine (13)
Yield 126 mg (56%), white solid.
MS (ESI$^+$): *m/z* 274 [M+H]$^+$, 272 [M-H]$^-$. UV ($CHCl_3$) λ_{max} 238; 275 nm.
^{1}H NMR (DMSO-d_6): δ = 3.47–3.60 (m, 2H, CH_2), 3.68 (s, 3H, CH_3), 3.79 (q, *J* = 3.4 Hz, 1H, CH), 3.93 (q, *J* = 4.6 Hz, 1H, CH), 3.98 (q, *J* = 5.8 Hz, 1H, CH), 4.98–5.07 (m, 2H, CH and OH), 5.26 (d, *J* = 5.9 Hz, 1H, OH), 5.57 (d, *J* = 8.2 Hz, 1H, CH=CH), 5.74 (d, *J* = 6.1 Hz, 1H, OH), 7.15 (d, *J* = 8.3 Hz, 1H, CH=CH), 9.87 (s, 1H, NH).
^{13}C NMR (DMSO-d_6): δ = 59.31, 59.64, 68.62, 70.93, 82.96, 85.22, 95.76, 129.60, 142.23, 147.67.

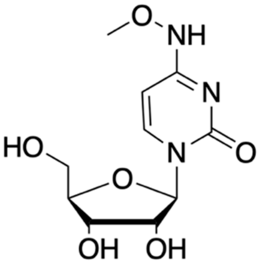

N^4-Ethoxycytidine (14)

Yield 134 mg (57%), white solid.
MS (ESI$^+$): *m/z* 288 [M+H]$^+$, 286 [M-H]$^-$. UV ($CHCl_3$) λ_{max} 241; 276 nm.
^{1}H NMR (DMSO-d_6): δ = 1.11–1.29 (m, 5H, CH_2 and CH3), 3.49–3.60 (m, 2H, 2CH), 3,92 (d, *J* = 6.8 Hz, 2H, CH_2), 3.96–4.01 (m, 1H, CH), 4.98–5.08 (m, 2H, CH and OH), 5.26 (d, *J* = 5.9 Hz, 1H, OH), 5.59 (d, *J* = 8.2 Hz, 1H, CH=CH), 5.74 (d, *J* = 6.1 Hz, 1H, OH), 7.14 (d, *J* = 8.2 Hz, 1H, CH=CH), 9.75 (s, 1H, NH).
^{13}C NMR (DMSO-d_6): δ = 14.90, 61.75, 68.72, 70.73, 73.03, 85.06, 87.29, 98.12, 131.53, 144.30, 149.83.

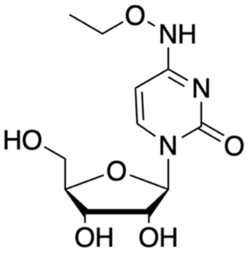

^{1}H and ^{13}C NMR Spectra

1-(3,4-Dihydroxy-5-(hydroxymethyl)oxolan-2-yl)-4-((2,4-dinitrophenyl)sulfanyl) pyrimidin-2(1H)-one

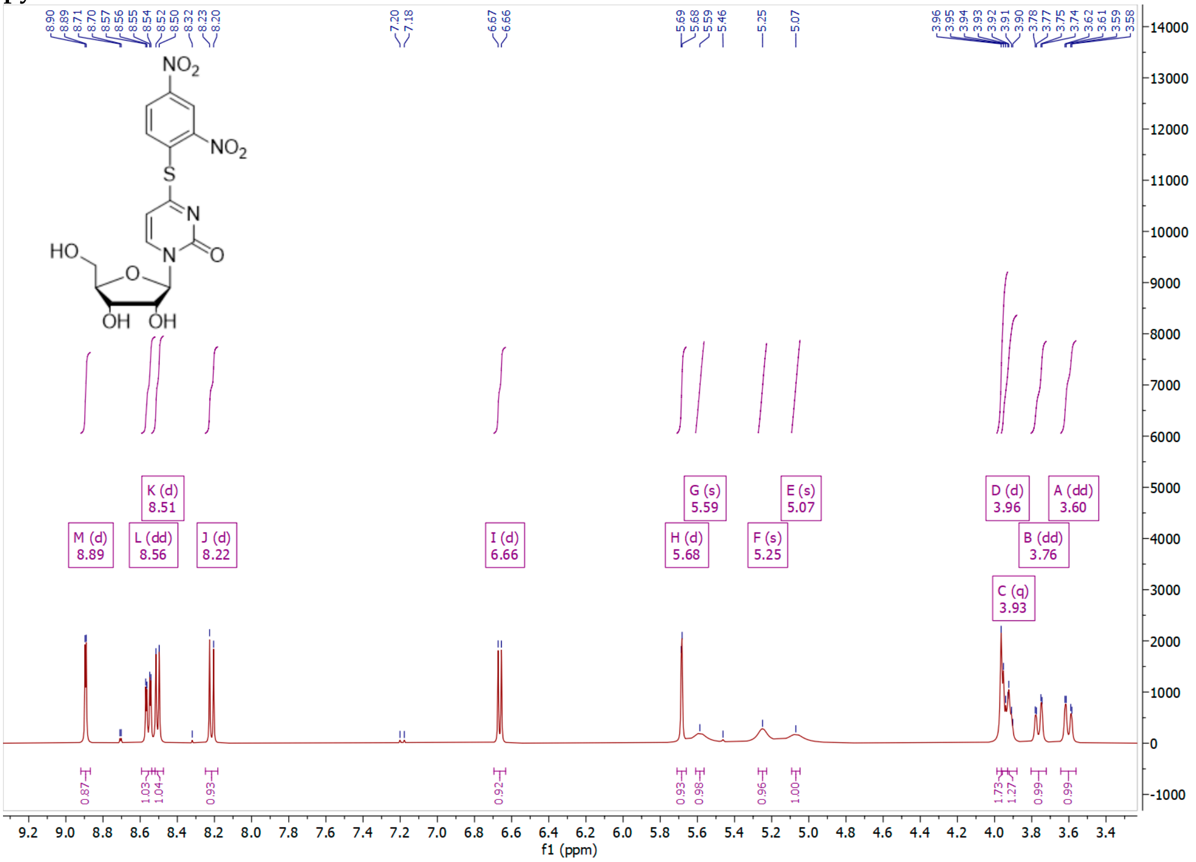

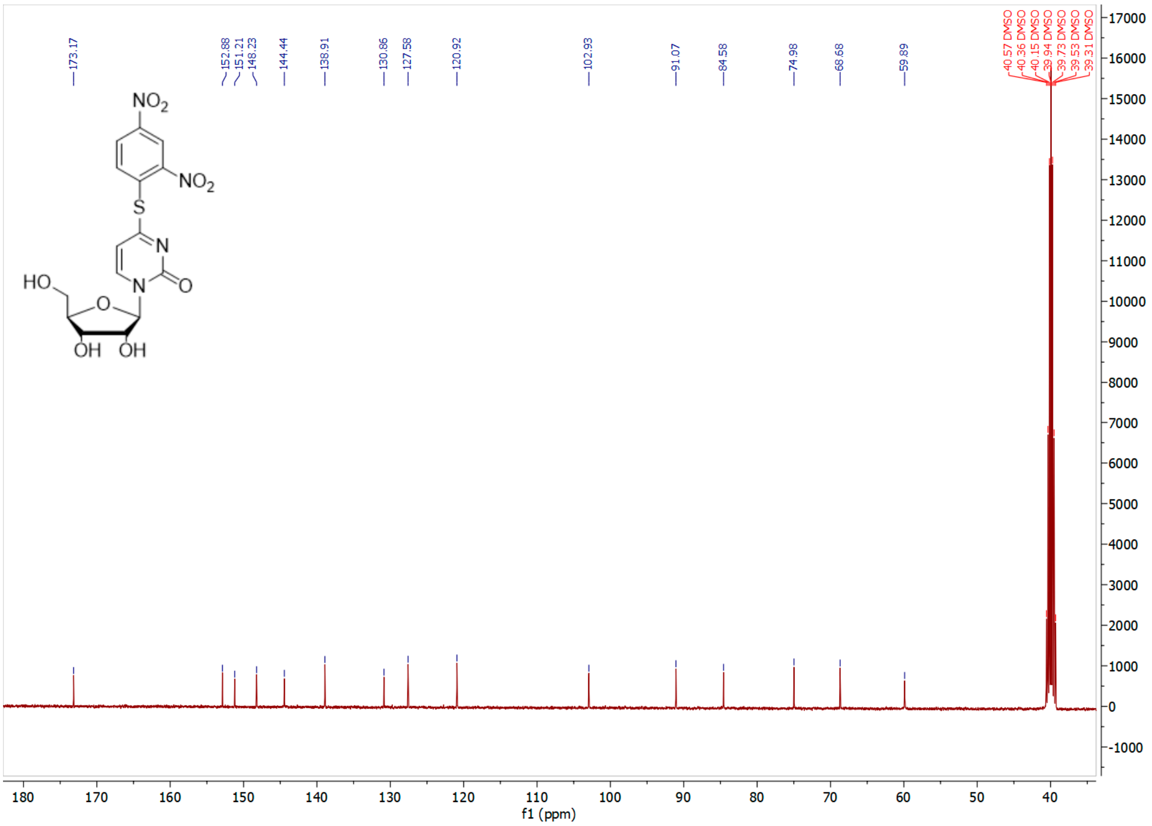

***N*-[1-(β-D-Ribofuranosyl)-2-oxo-4-pyrimidinyl]-glycine (10)**

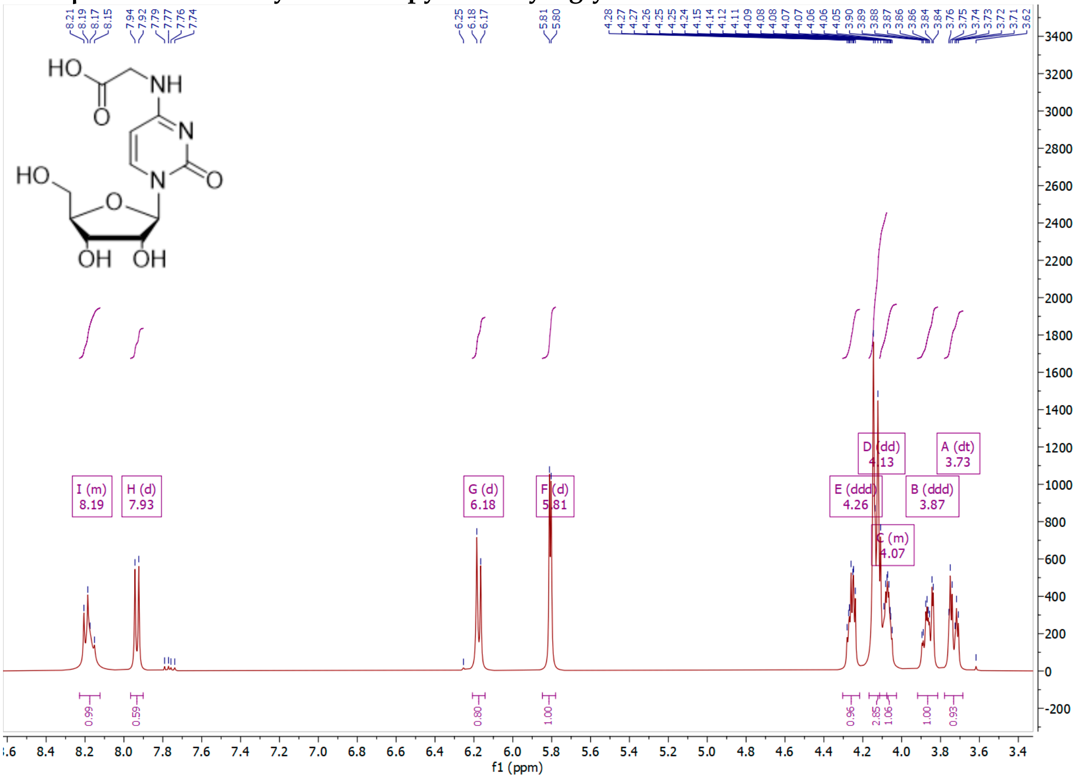

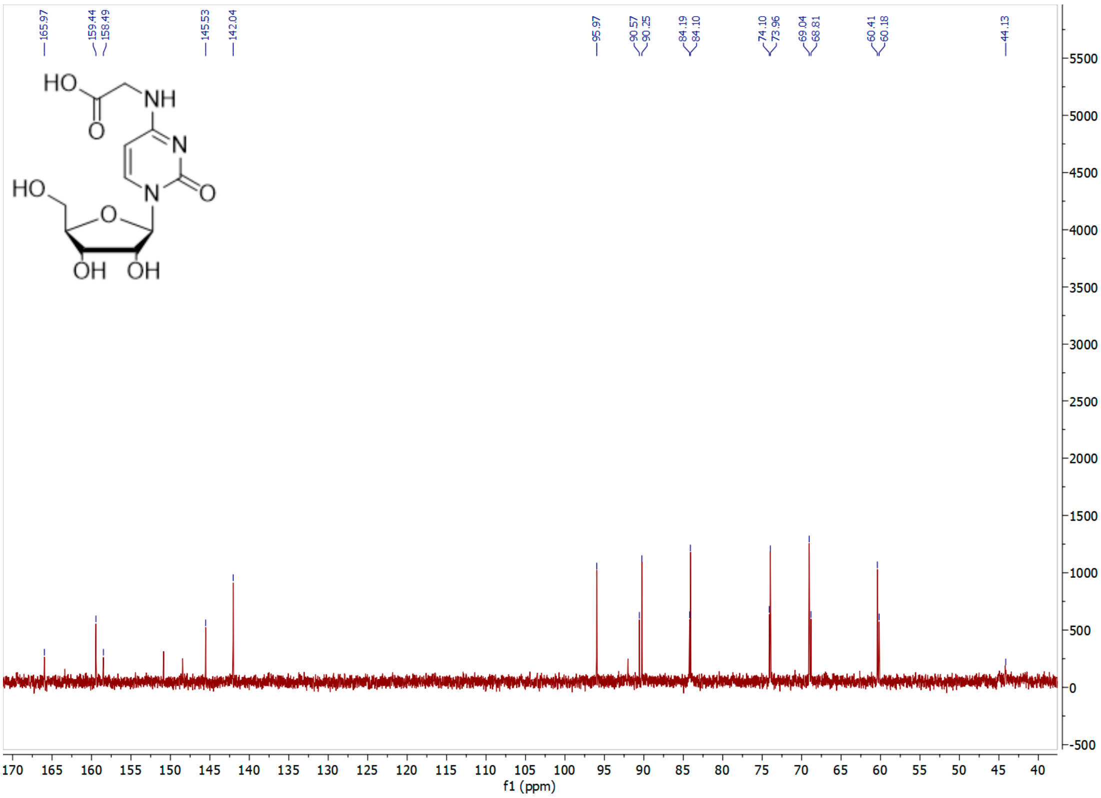

N-[1-(β-D-Ribofuranosyl)-2-oxo-4-pyrimidinyl]-L-alanine (11)

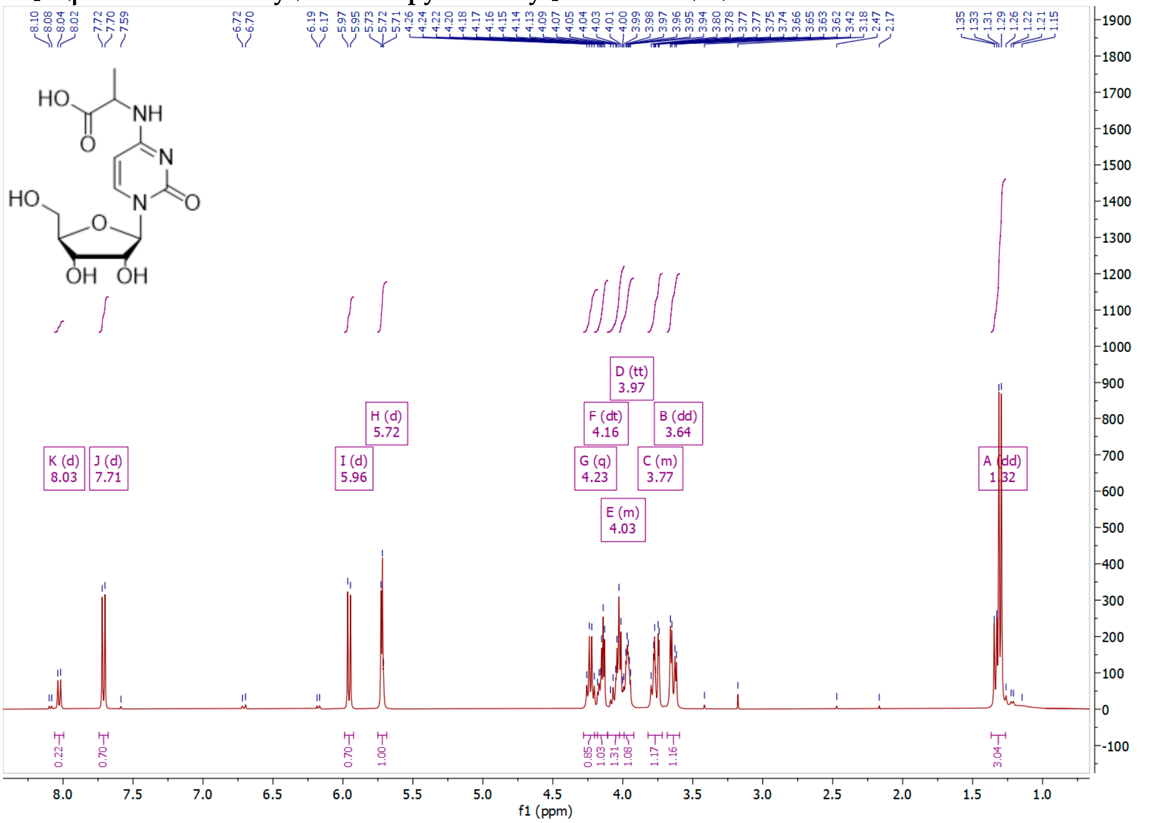

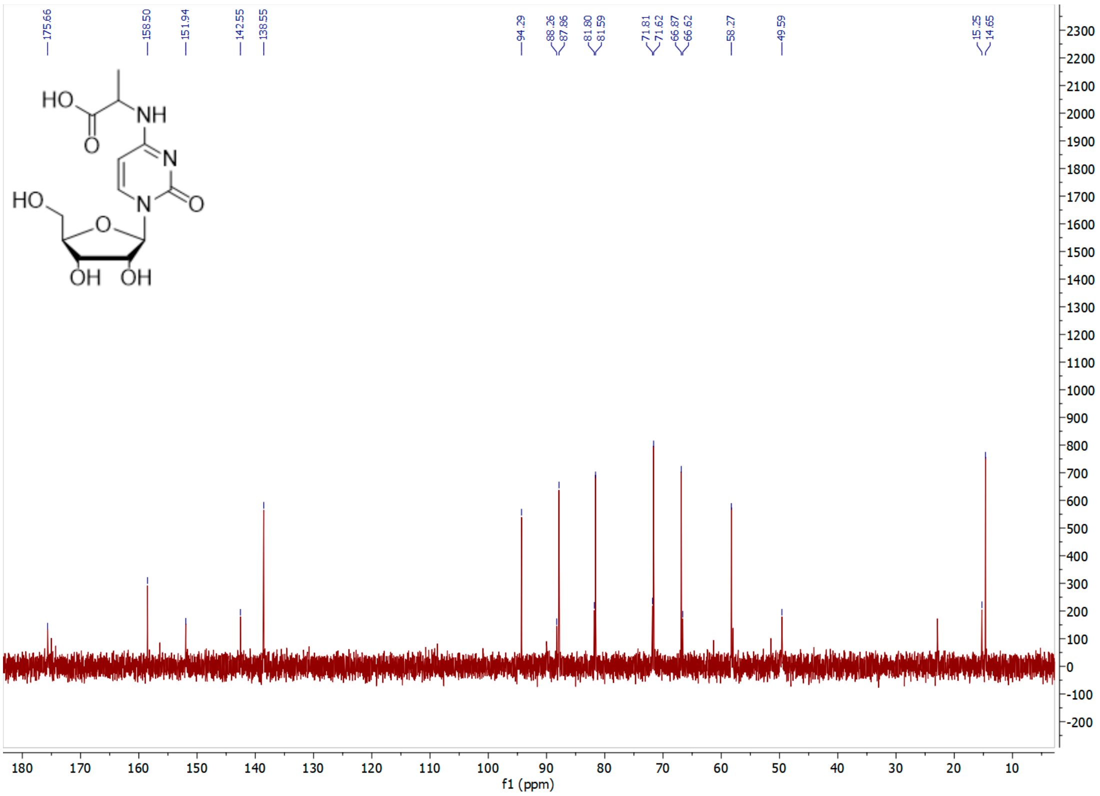

N^4-Hydroxycytidine (12)

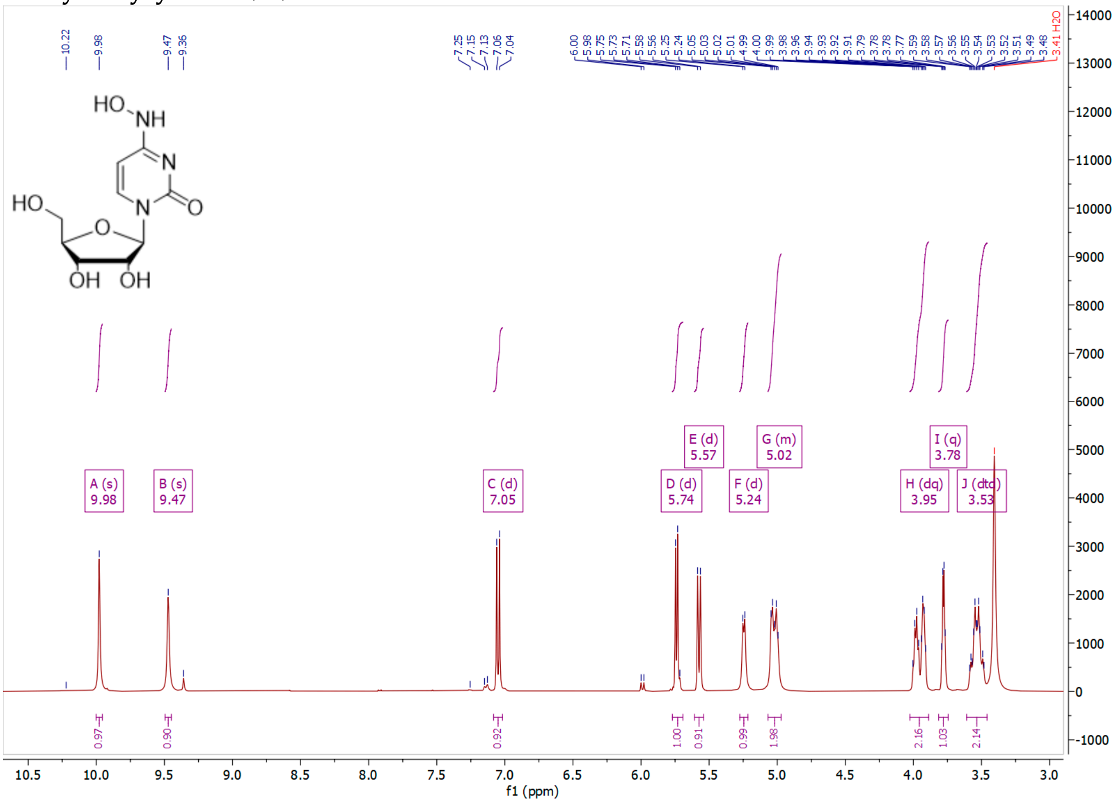

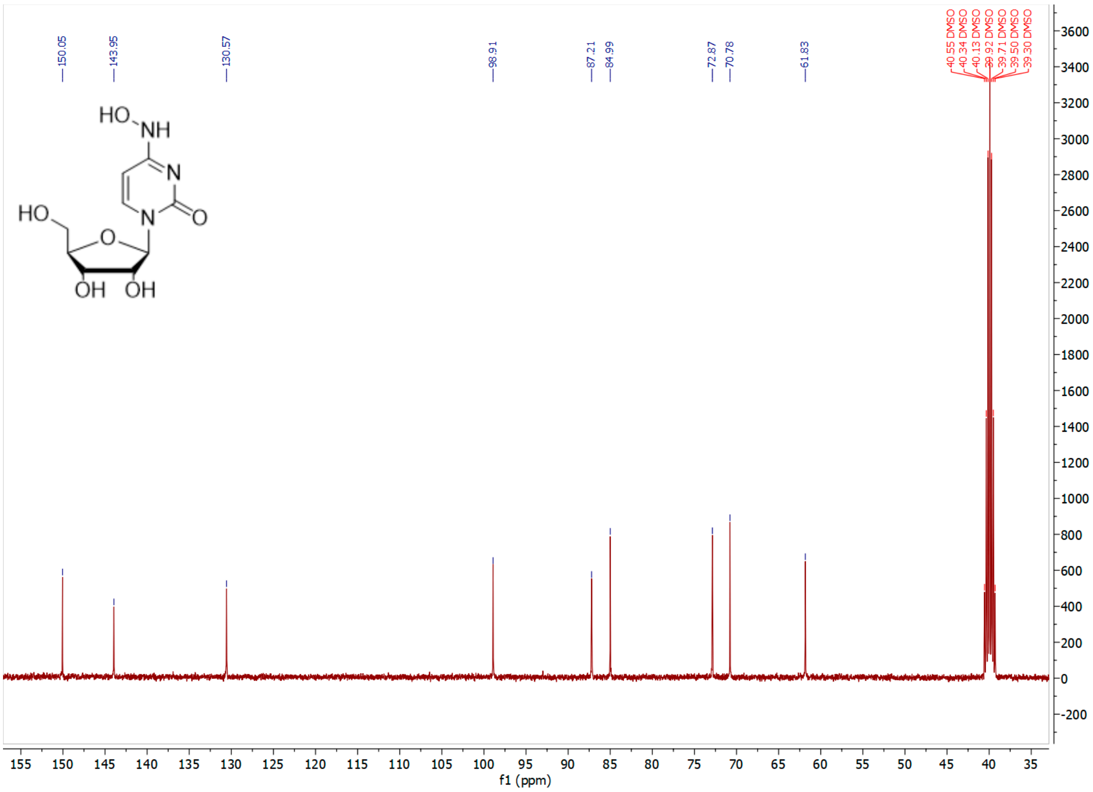

N^4-Methoxycytidine (13)

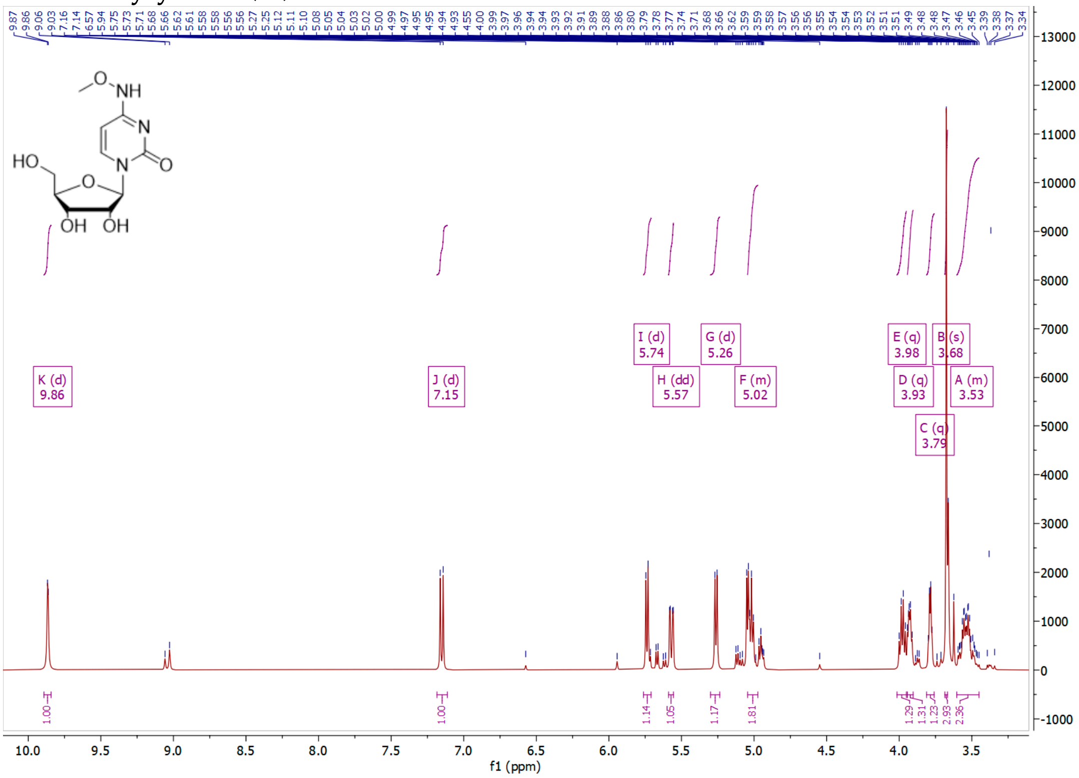

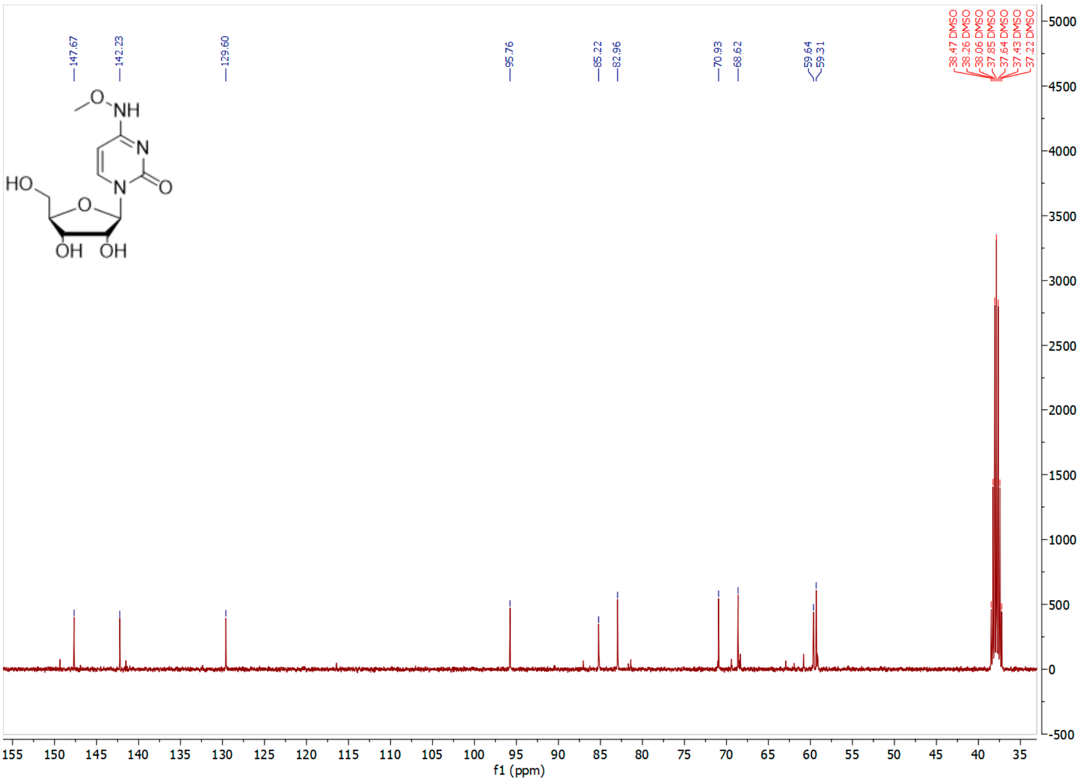

N[4]-Ethoxycytidine (14)

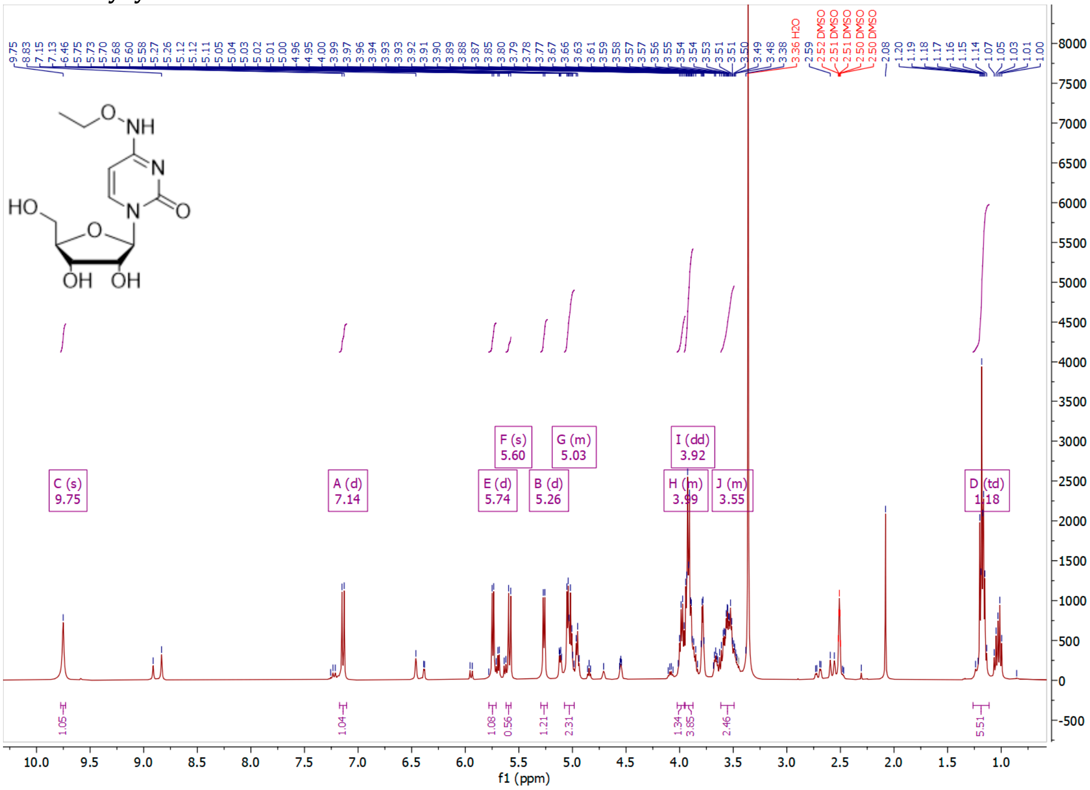

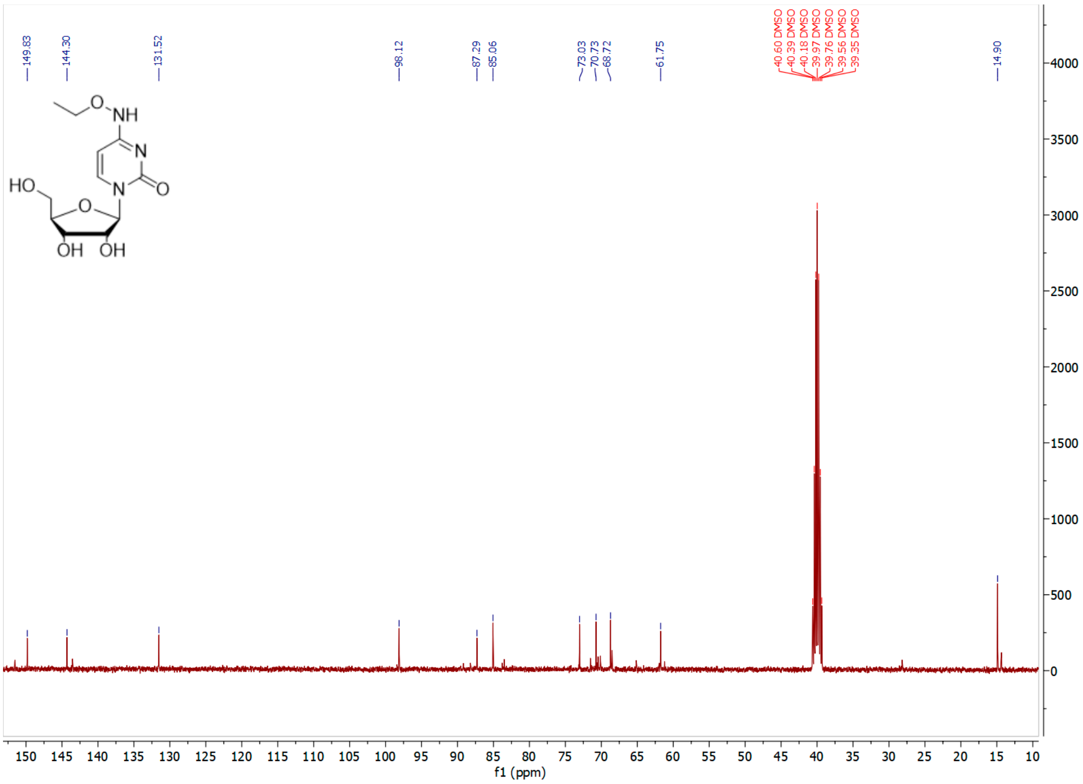

MS spectra

1-(3,4-Dihydroxy-5-(hydroxymethyl)oxolan-2-yl)-4-((2,4-dinitrophenyl)sulfanyl) pyrimidin-2(1H)-one

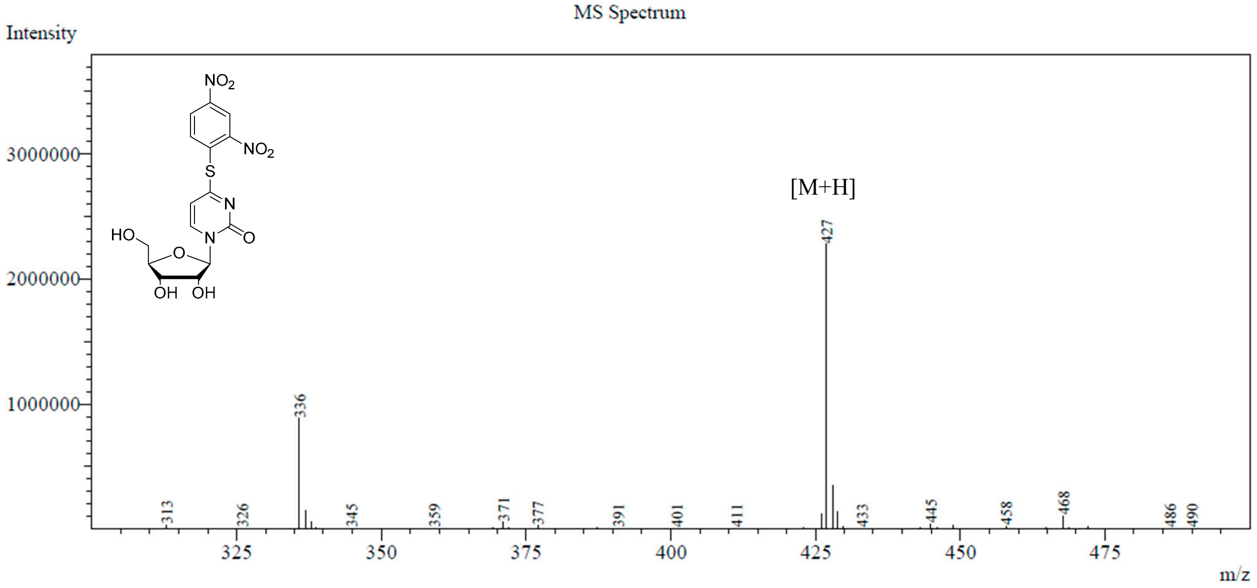

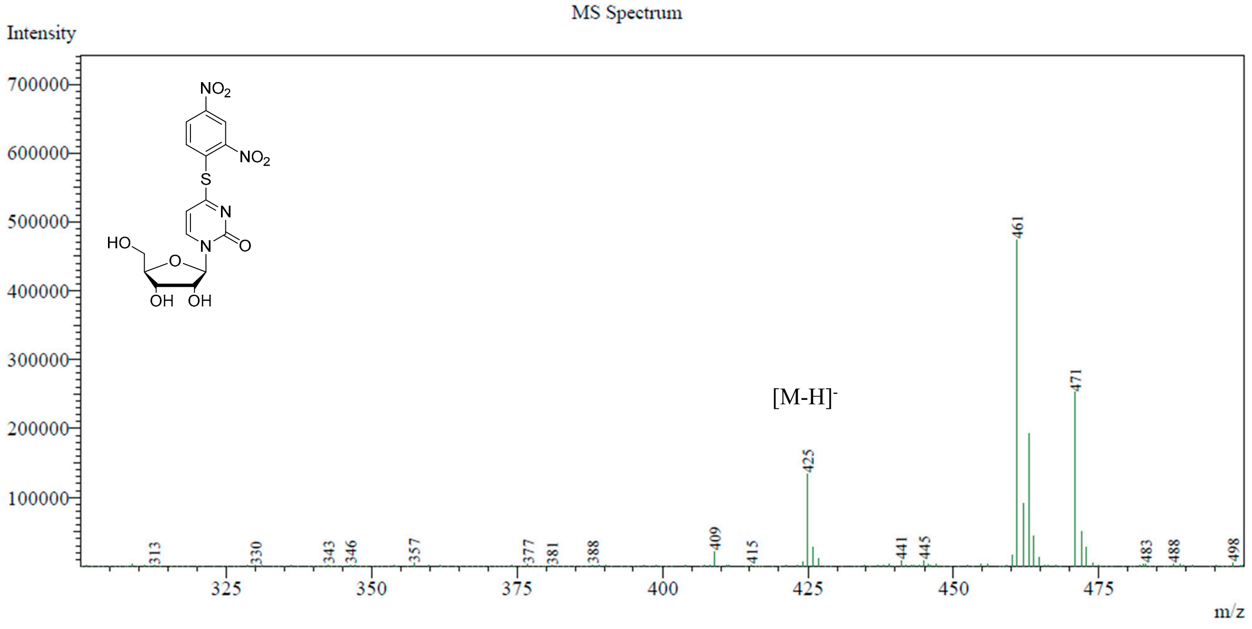

***N*-[1-(β-D-Ribofuranosyl)-2-oxo-4-pyrimidinyl]-glycine (10)**

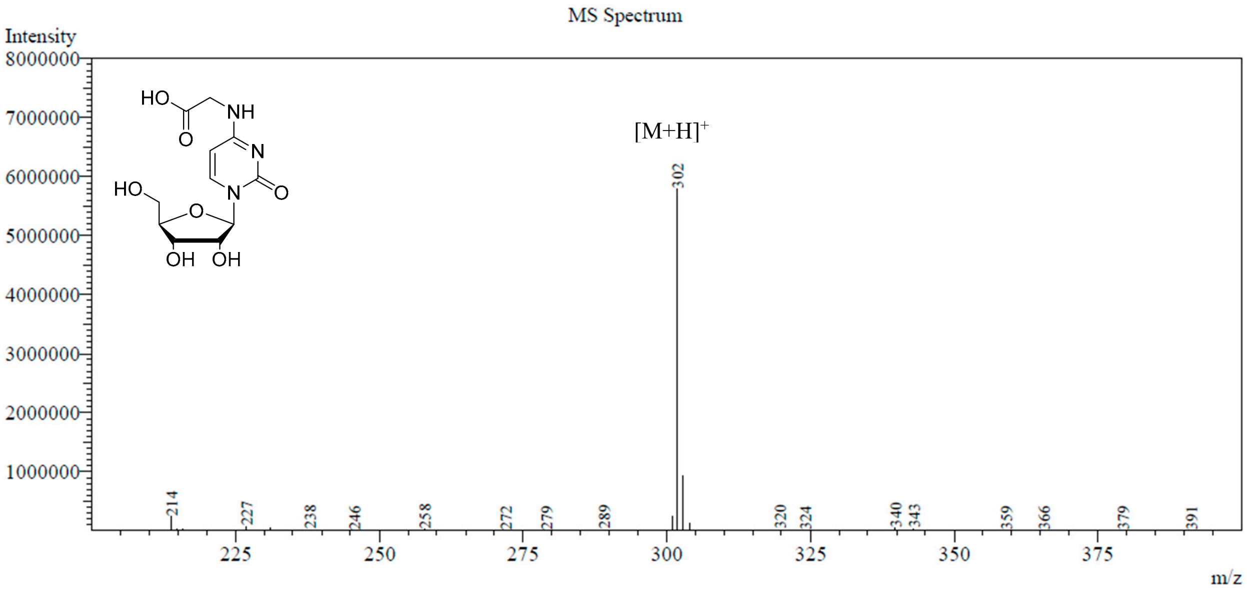

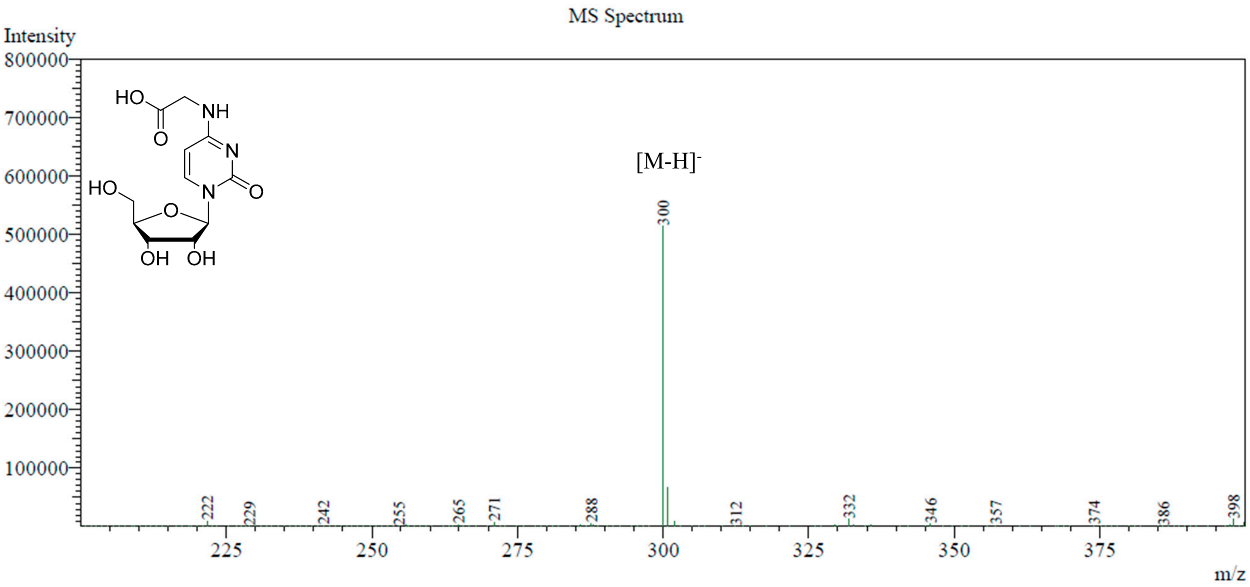

***N*-[1-(β-D-Ribofuranosyl)-2-oxo-4-pyrimidinyl]-L-alanine (11)**

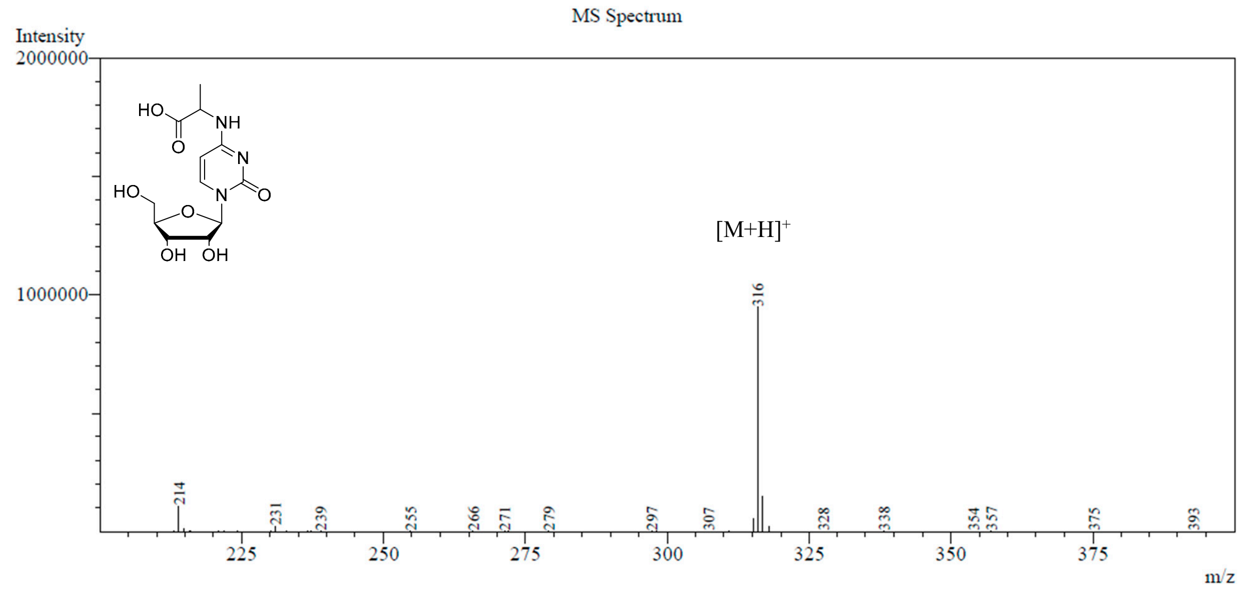

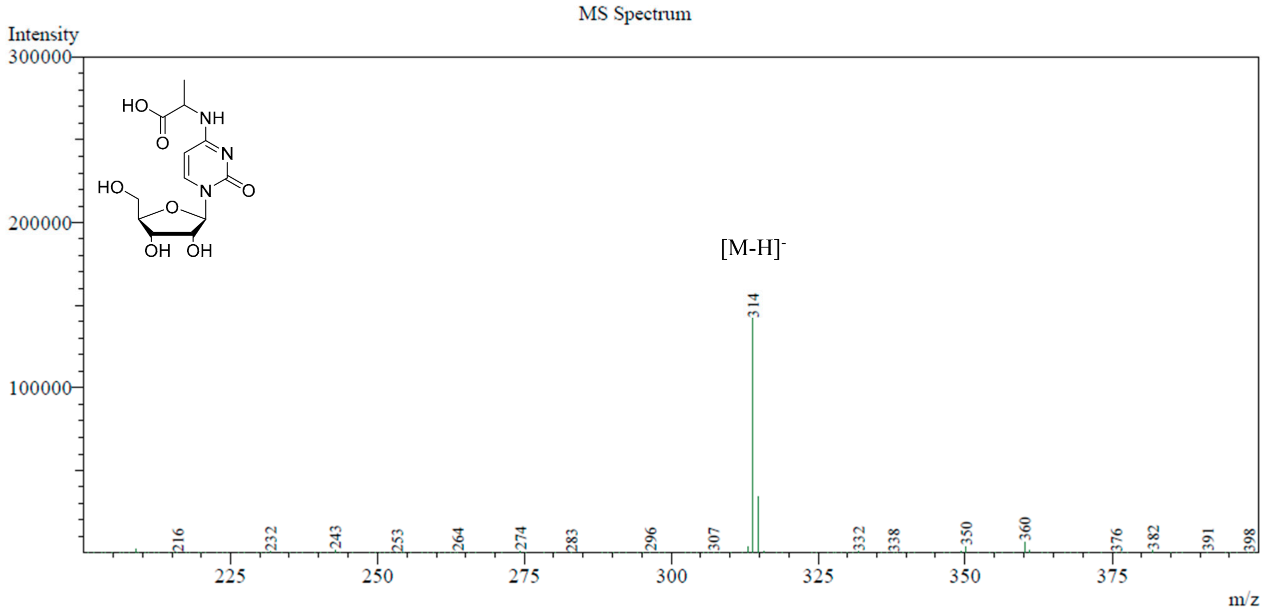

***N*[4]-Hydroxycytidine (12)**

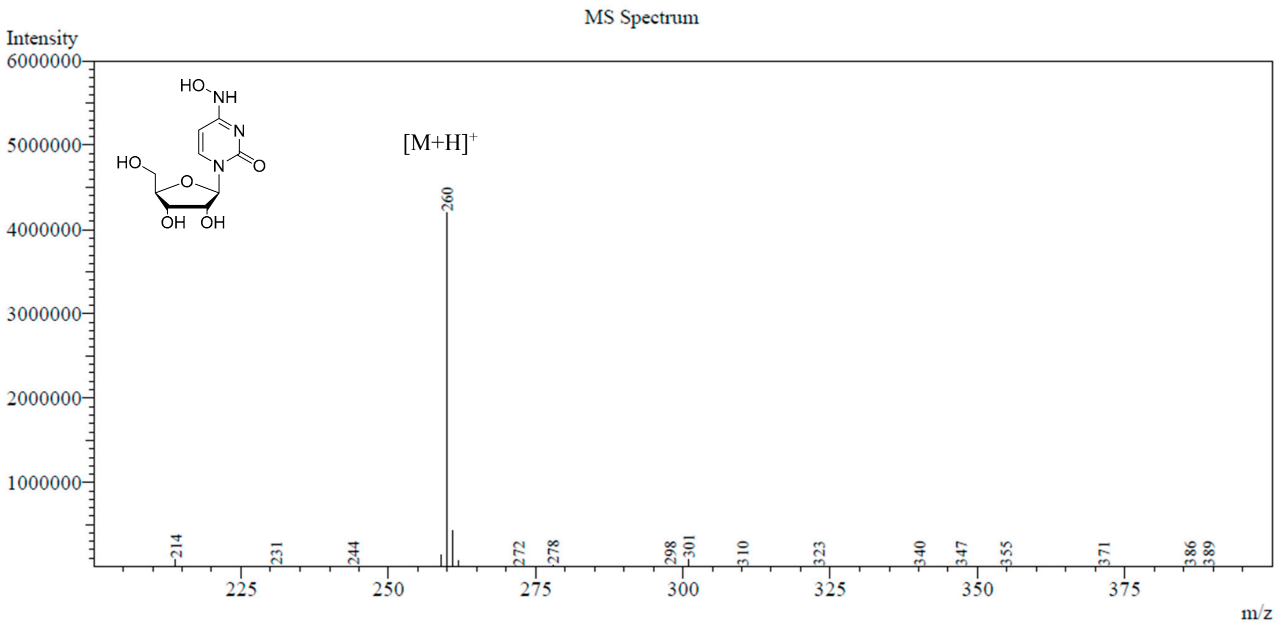

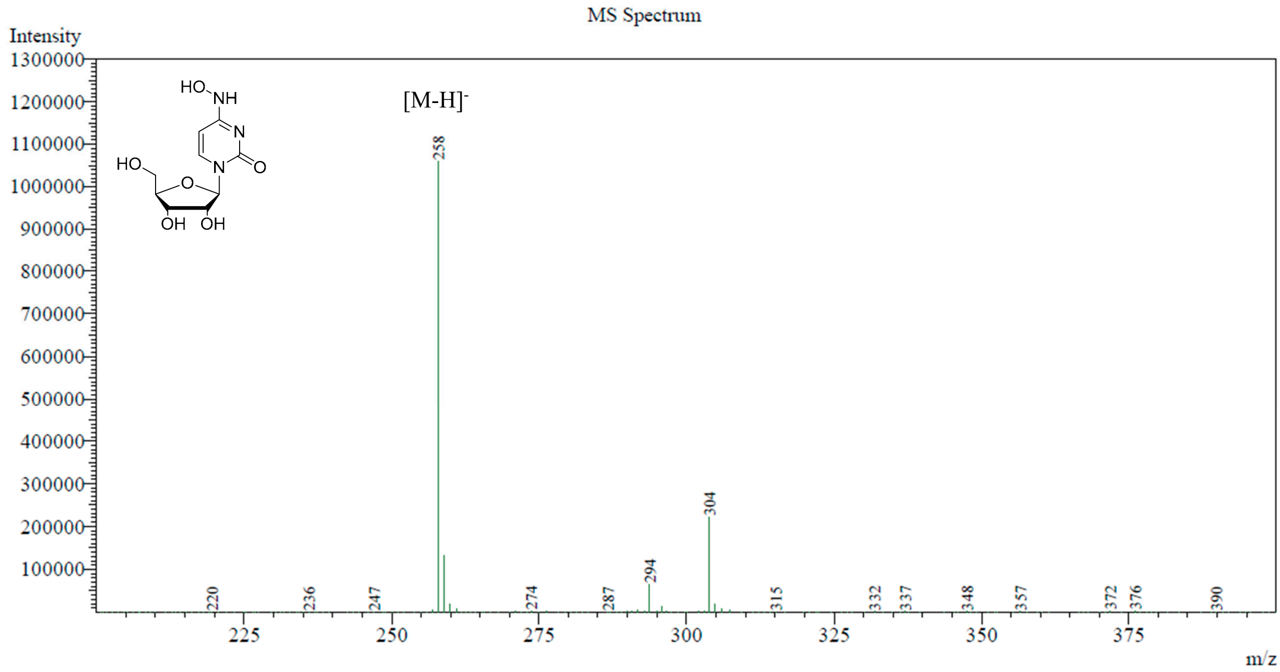

N^4-Methoxycytidine (13)

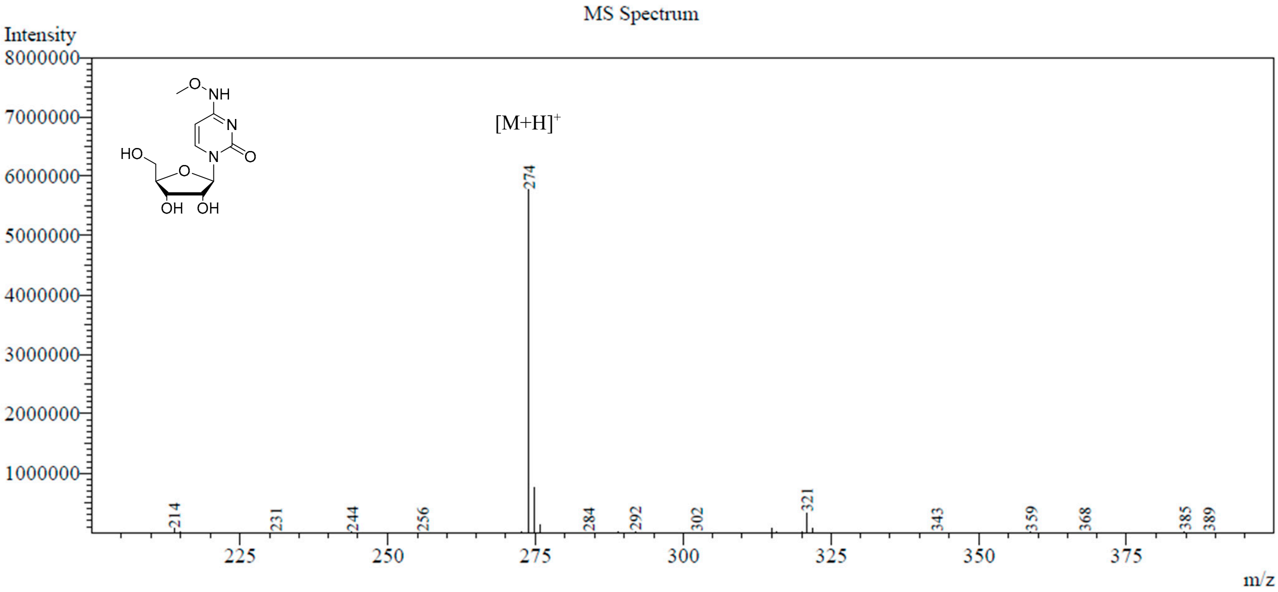

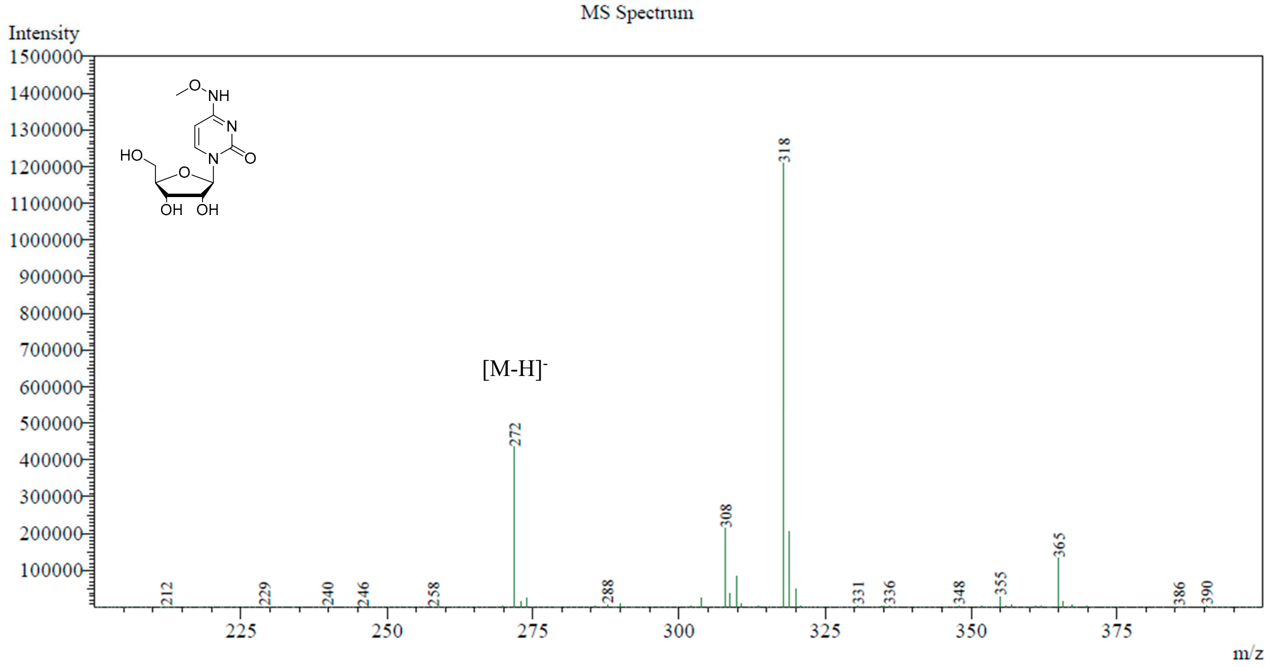

N^4-Ethoxycytidine (14)

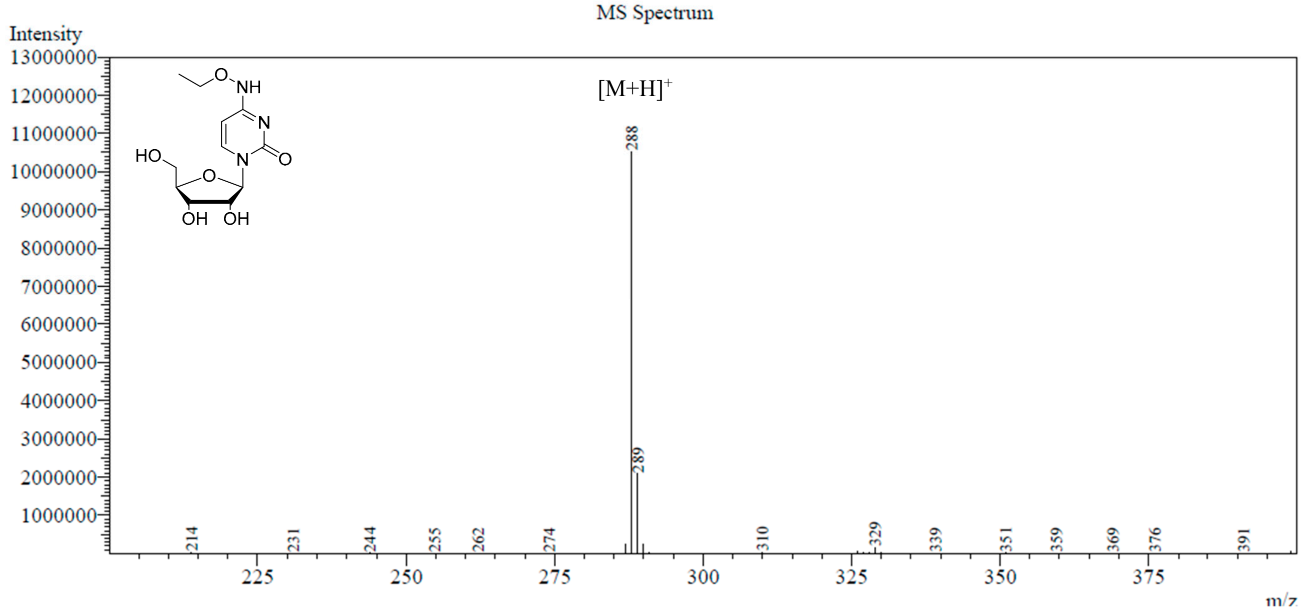

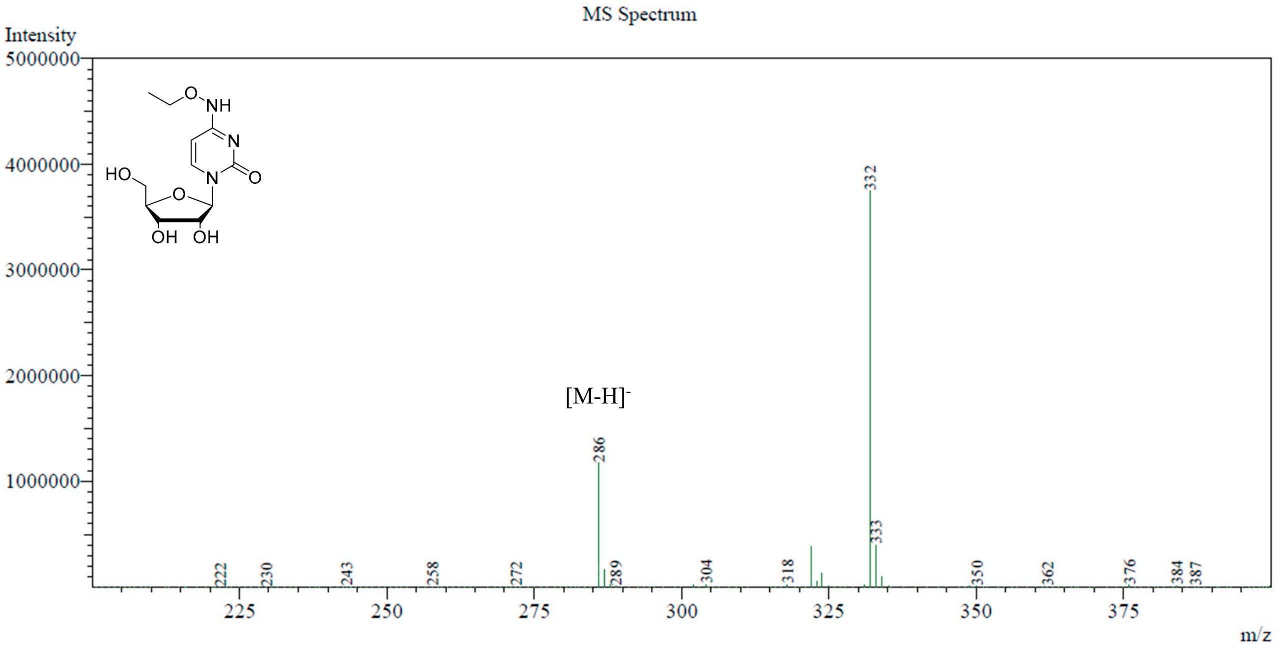

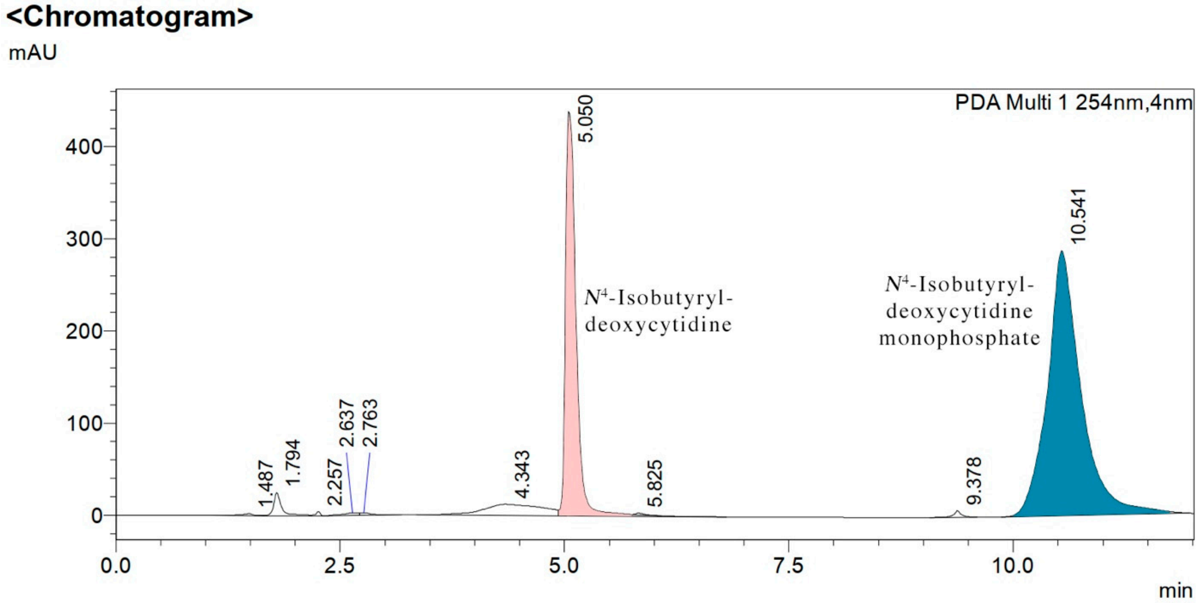

Figure S1. HPLC chromatogram of *Dm*dNK-V84A catalysed N^4-isobutyryl-deoxycytidine phosphorylation reaction. Pink peak – nucleoside area, blue peak – nucleoside monophosphate area.

Phosphorylation efficiencies (%) were calculated using equation: $100\% \times \frac{NMP\ area}{Nucleoside\ area + NMP\ area}$

Table S2. HPLC data of *Dm*dNK catalysed reactions.

Substrate	*Dm*dNK	Peak area		Phosphorylation efficiency, %
		Nucleoside	NMP	
**1** (2'-Deoxycytidine)	Without kinase	3367613	0	0.0
	WT	92593	3763377	97.6
	W57F	394247	3329378	89.4
	W57V	392142	3146097	88.9
	Q81A	420285	3147839	88.2
	Q81A+V84G	247227	2831374	92.0
	Q81A+M88G	214191	3083438	93.5
	Q81A+A110G	210801	2931223	93.3
	V84A	61402	1806335	96.7
	V84A+M88A	112444	3759948	97.1
	V84A+A110D	1826111	517291	22.1
	V84G	427860	3168249	88.1
	M88A	664806	2465678	78.8
	M88G	458085	3116787	87.2
	M88R	2901433	1466319	33.6
	M88R+A110G	427621	4445858	91.2

	A110D	192126	4541214	95.9
	A110G	349427	2837025	89.0
**2** (Cytidine)	Without kinase	4087340	0	0.0
	WT	552371	1975613	78.1
	W57F	1758163	1811540	50.7
	W57V	3885669	226941	5.5
	Q81A	3609045	587377	14.0
	Q81A+V84G	3386114	81021	2.3
	Q81A+M88G	3705134	170467	4.4
	Q81A+A110G	3035086	454723	13.0
	V84A	1210956	3537727	74.5
	V84A+M88A	1492296	3615341	70.8
	V84A+A110D	5629205	29859	0.5
	V84G	2485850	1693511	40.5
	M88A	2435910	2148976	46.9
	M88G	2536147	1085993	30.0
	M88R	272416	13087	4.6
	M88R+A110G	2501241	102118	3.9
	A110D	4728591	500150	9.6
	A110G	1299777	2396103	64.8
**3** (*N*[4]-Glycinoyl)-2'-deoxycytidine	Without kinase	3533920	0	0.0
	WT	1766625	779115	30.6
	W57F	4481985	53803	1.2
	W57V	3921649	14319	0.4
	Q81A	2914894	734726	20.1
	Q81A+V84G	507497	2305311	82.0
	Q81A+M88G	2897500	216329	6.9
	Q81A+A110G	40365	2551153	98.4
	V84A	257161	5279565	95.4
	V84A+M88A	1021945	1555364	60.3
	V84A+A110D	5896283	0	0.0
	V84G	63370	3082212	98.0
	M88A	4426277	740592	14.3
	M88G	3374973	166883	4.7
	M88R	3090402	20866	0.7
	M88R+A110G	8205490	0	0.0
	A110D	4436973	562039	11.2
	A110G	61842	2878933	97.9

**4** (*N*[4]-Boc-Glycinoyl)-2'-deoxycytidine	Without kinase	5830736	0	0.0
	WT	2076475	0	0.0
	W57F	6250263	0	0.0
	W57V	6277554	0	0.0
	Q81A	5935932	0	0.0
	Q81A+V84G	5016231	0	0.0
	Q81A+M88G	5251527	0	0.0
	Q81A+A110G	4428507	0	0.0
	V84A	1199141	0	0.0
	V84A+M88A	1352116	0	0.0
	V84A+A110D	1173236	0	0.0
	V84G	6335900	0	0.0
	M88A	4075704	0	0.0
	M88G	6251443	0	0.0
	M88R	1158916	0	0.0
	M88R+A110G	1226345	0	0.0
	A110D	1181149	0	0.0
	A110G	9017496	0	0.0
**5** (*N*[4]-Alaninoyl)-2'-deoxycytidine	Without kinase	4204377	0	0.0
	WT	1727193	50107	2.8
	W57F	3781874	0	0.0
	W57V	3880622	0	0.0
	Q81A	4076282	0	0.0
	Q81A+V84G	2509985	1122337	30.9
	Q81A+M88G	3984573	38355	1.0
	Q81A+A110G	1623436	1954088	54.6
	V84A	1019137	3095688	75.2
	V84A+M88A	1866003	2405183	56.3
	V84A+A110D	4898880	0	0.0
	V84G	554900	3011397	84.4
	M88A	3635063	47459	1.3
	M88G	3954720	40647	1.0
	M88R	2439032	0	0.0
	M88R+A110G	2130654	0	0.0
	A110D	203341	0	0.0
	A110G	2238621	1093461	32.8
	Without kinase	5050205	0	0.0
	WT	1602599	0	0.0

**6**<br>(N^4-Boc-Alaninoyl)-2'-deoxycytidine	W57F	5578115	0	0.0
	W57V	5239237	0	0.0
	Q81A	5219399	0	0.0
	Q81A+V84G	5800200	0	0.0
	Q81A+M88G	5999875	0	0.0
	Q81A+A110G	5260184	0	0.0
	V84A	1722611	0	0.0
	V84A+M88A	1602950	0	0.0
	V84A+A110D	1169720	0	0.0
	V84G	5583289	0	0.0
	M88A	5327046	0	0.0
	M88G	5363911	0	0.0
	M88R	1453016	0	0.0
	M88R+A110G	1575993	0	0.0
	A110D	1569294	0	0.0
	A110G	5009856	0	0.0
**7**<br>(N^4-Boc-Leucinoyl)-2'-deoxycytidine	Without kinase	1489525	0	0.0
	WT	2063008	0	0.0
	W57F	1054552	0	0.0
	W57V	1661980	0	0.0
	Q81A	1608730	0	0.0
	Q81A+V84G	1702345	0	0.0
	Q81A+M88G	1701975	0	0.0
	Q81A+A110G	1713971	0	0.0
	V84A	1681002	0	0.0
	V84A+M88A	555225	0	0.0
	V84A+A110D	1610472	0	0.0
	V84G	1642071	0	0.0
	M88A	4682872	0	0.0
	M88G	1096747	0	0.0
	M88R	1600828	0	0.0
	M88R+A110G	1296376	0	0.0
	A110D	2580586	0	0.0
	A110G	1669809	0	0.0
**8**<br>(N^4-Acetyl-2'-deoxycytidine)	Without kinase	6226421	0	0.0
	WT	0	7011934	100.0
	W57F	4774506	1727663	26.6
	W57V	7051361	300275	4.1

	Q81A	0	6079533	100.0
	Q81A+V84G	862381	6316584	88.0
	Q81A+M88G	423805	5799208	93.2
	Q81A+A110G	0	6424772	100.0
	V84A	0	1569910	100.0
	V84A+M88A	0	7413202	100.0
	V84A+A110D	1064277	0	0.0
	V84G	0	5257480	100.0
	M88A	9044240	10507882	53.7
	M88G	4745506	1950387	29.1
	M88R	1156029	0	0.0
	M88R+A110G	1041064	0	0.0
	A110D	5734730	2141464	27.2
	A110G	0	6354837	100.0
**9** (N^4-Isobutyryl-2'-deoxycytidine)	Without kinase	8336139	0	0.0
	WT	3742183	0	0.0
	W57F	7965525	65236	0.8
	W57V	8582852	18275	0.2
	Q81A	8682531	817324	8.6
	Q81A+V84G	3955767	6151724	60.9
	Q81A+M88G	8298631	234154	2.7
	Q81A+A110G	1114525	7660912	87.3
	V84A	3574294	7499356	67.7
	V84A+M88A	2045768	0	0.0
	V84A+A110D	4857652	0	0.0
	V84G	208974	3217997	93.9
	M88A	20438052	860276	4.0
	M88G	7817140	137176	1.7
	M88R	4820018	0	0.0
	M88R+A110G	4491418	0	0.0
	A110D	4743576	0	0.0
	A110G	5576191	2357616	29.7
**10** (*N*-[1-(β-D-Ribofuranosyl)-2-oxo-4-pyrimidinyl]-glycine)	Without kinase	2095327	0	0.0
	WT	1851147	0	0.0
	W57F	3028727	0	0.0
	W57V	2231023	0	0.0
	Q81A	3261361	0	0.0
	Q81A+V84G	2979180	0	0.0

	Q81A+M88G	2213667	0	0.0
	Q81A+A110G	1638266	0	0.0
	V84A	2852846	0	0.0
	V84A+M88A	2776665	0	0.0
	V84A+A110D	2885570	0	0.0
	V84G	2693111	0	0.0
	M88A	2615862	0	0.0
	M88G	2250385	0	0.0
	M88R	2899988	0	0.0
	M88R+A110G	3165145	0	0.0
	A110D	3032285	0	0.0
	A110G	2594869	0	0.0
**11** (*N*-[1-(β-D-Ribofuranosyl)-2-oxo-4-pyrimidinyl]-L-alanine)	Without kinase	833571	0	0.0
	WT	734802	0	0.0
	W57F	734918	0	0.0
	W57V	988460	0	0.0
	Q81A	993914	0	0.0
	Q81A+V84G	826495	0	0.0
	Q81A+M88G	73563	0	0.0
	Q81A+A110G	743893	0	0.0
	V84A	966336	0	0.0
	V84A+M88A	941699	0	0.0
	V84A+A110D	972812	0	0.0
	V84G	977768	0	0.0
	M88A	742642	0	0.0
	M88G	8077777	0	0.0
	M88R	913571	0	0.0
	M88R+A110G	949771	0	0.0
	A110D	925441	0	0.0
	A110G	999994	0	0.0
**12** (*N*^{4}-Hydroxycytidine)	Without kinase	6957218	0	0.0
	WT	4581853	3861607	45.7
	W57F	5437221	360578	6.2
	W57V	6196138	0	0.0
	Q81A	5972921	129112	2.1
	Q81A+V84G	4762798	0	0.0
	Q81A+M88G	4970905	0	0.0
	Q81A+A110G	4174294	514869	11.0

	V84A	6524433	2063609	24.0
	V84A+M88A	9399391	124324	1.3
	V84A+A110D	9908937	0	0.0
	V84G	6139909	245118	3.8
	M88A	9569047	994710	9.4
	M88G	5679863	338782	5.6
	M88R	9527989	0	0.0
	M88R+A110G	10154406	0	0.0
	A110D	9330771	0	0.0
	A110G	5483570	449989	7.6
**13** (N^4-Methoxycytidine)	Without kinase	6298798	0	0.0
	WT	8527638	482651	5.4
	W57F	6563009	0	0.0
	W57V	8186247	0	0.0
	Q81A	5513541	690127	11.1
	Q81A+V84G	6075099	230225	3.7
	Q81A+M88G	5900204	359041	5.7
	Q81A+A110G	4921032	2099846	29.9
	V84A	7865542	664750	7.8
	V84A+M88A	7295730	234018	3.1
	V84A+A110D	8701616	0	0.0
	V84G	6062395	362182	5.6
	M88A	7868375	0	0.0
	M88G	6530964	0	0.0
	M88R	7844108	0	0.0
	M88R+A110G	8288039	0	0.0
	A110D	8376945	0	0.0
	A110G	5889469	229620	3.8
**14** (N^4-Ethoxycytidine)	Without kinase	5478738	0	0.0
	WT	7238436	7852	0.1
	W57F	7595066	0	0.0
	W57V	5134201	0	0.0
	Q81A	5088628	187290	3.5
	Q81A+V84G	5560400	186653	3.2
	Q81A+M88G	5306972	50236	0.9
	Q81A+A110G	3684938	1568610	29.9
	V84A	6696433	129283	1.9
	V84A+M88A	7493848	128758	1.7

	V84A+A110D	7934995	0	0.0
	V84G	5461226	103105	1.9
	M88A	5446241	0	0.0
	M88G	5514336	0	0.0
	M88R	7682104	0	0.0
	M88R+A110G	7692127	0	0.0
	A110D	6733323	0	0.0
	A110G	5499657	16228	0.3

Table S3. HPLC data of *Bs*dCK catalysed reactions.

Substrate	*Bs*dCK	Peak area		Phosphorylation efficiency, %
		Nucleoside	NMP	
**1** (2'-Deoxycytidine)	Without kinase	2930399	0	0.0
	WT	0	2888740	100.0
	R70M	2787613	216498	7.2
	R70M+D93A	0	2322814	100.0
	D93A	0	2828597	100.0
**2** (Cytidine)	Without kinase	3591594	0	0.0
	WT	296785	3880122	92.9
	R70M	3722431	76054	2.0
	R70M+D93A	1341955	1661685	55.3
	D93A	3490442	179443	4.9
**3** (N^4-Glycinoyl)-2'-deoxycytidine	Without kinase	3039473	0	0.0
	WT	2804760	352224	11.2
	R70M	3567144	79876	2.2
	R70M+D93A	38323	2177564	98.3
	D93A	2463346	606523	19.8
**4** (N^4-Boc-Glycinoyl)-2'-deoxycytidine	Without kinase	5136603	0	0.0
	WT	1088156	0	0.0
	R70M	2519799	0	0.0
	R70M+D93A	2356092	0	0.0
	D93A	3971322	0	0.0
**5** (N^4-Alaninoyl)-2'-deoxycytidine	Without kinase	4331177	0	0.0
	WT	4009864	0	0.0
	R70M	4558531	0	0.0
	R70M+D93A	2388195	1408877	37.1
	D93A	3281109	630759	16.1

**6** (N^4-Boc-Alaninoyl)-2'-deoxycytidine	Without kinase	5318794	0	0.0
	WT	1876946	0	0.0
	R70M	2381617	0	0.0
	R70M+D93A	1545513	0	0.0
	D93A	5018990	0	0.0
**7** (N^4-Boc-Leucinoyl)-2'-deoxycytidine	Without kinase	2528440	0	0.0
	WT	694394	0	0.0
	R70M	2411274	0	0.0
	R70M+D93A	1938504	0	0.0
	D93A	2226369	0	0.0
**8** (N^4-Acetyl-2'-deoxycytidine)	Without kinase	5469333	0	0.0
	WT	328800	6705399	95.3
	R70M	5789481	0	0.0
	R70M+D93A	48490	6003835	99.2
	D93A	173266	5947462	97.2
**9** (N^4-Isobutyryl-2'-deoxycytidine)	Without kinase	5822802	0	0.0
	WT	6382643	146473	2.2
	R70M	6832100	0	0.0
	R70M+D93A	191263	7484473	97.5
	D93A	1639796	6279773	79.3
**10** (*N*-[1-(β-D-Ribofuranosyl)-2-oxo-4-pyrimidinyl]-glycine)	Without kinase	2095327	0	0.0
	WT	2469729	0	0.0
	R70M	1797050	0	0.0
	R70M+D93A	2939185	0	0.0
	D93A	2552524	0	0.0
**11** (*N*-[1-(β-D-Ribofuranosyl)-2-oxo-4-pyrimidinyl]-L-alanine)	Without kinase	833571	0	0.0
	WT	946904	0	0.0
	R70M	941187	0	0.0
	R70M+D93A	863948	0	0.0
	D93A	927565	0	0.0
**12** (N^4-Hydroxycytidine)	Without kinase	4956044	0	0.0
	WT	4135902	895245	17.8
	R70M	5124320	0	0.0
	R70M+D93A	4369464	416787	8.7
	D93A	5051431	108479	2.1
**13** (N^4-Methoxycytidine)	Without kinase	5569813	0	0.0
	WT	5743863	63138	1.1
	R70M	6057452	0	0.0
	R70M+D93A	5298561	0	0.0

	D93A	5668908	0	0.0
**14** (N^4-Ethoxycytidine)	Without kinase	4811451	0	0.0
	WT	4943009	381514	7.2
	R70M	5136084	0	0.0
	R70M+D93A	5163063	24099	0.5
	D93A	4766512	0	0.0

References

[1] M. Koplūnaitė, K. Butkutė, R. Meškys, D. Tauraitė, Synthesis of Pyrimidine Nucleoside and Amino Acid Conjugates *Tetrahedron Lett* **2020**, *61*, 152598.
